# Digits in a dish: An *in vitro* system to assess the molecular genetics of hand/foot development at single-cell resolution

**DOI:** 10.3389/fcell.2023.1135025

**Published:** 2023-03-13

**Authors:** Allison M. Fuiten, Yuki Yoshimoto, Chisa Shukunami, H. Scott Stadler

**Affiliations:** ^1^ Research Center, Shriners Children’s, Portland, OR, United States; ^2^ Department of Orthopaedics and Rehabilitation, Oregon Health and Science University, Portland, OR, United States; ^3^ Department of Molecular Biology and Biochemistry, Graduate School of Biomedical and Health Sciences, Hiroshima University, Hiroshima, Japan

**Keywords:** *in vitro*, autopod development, joint development, single cell RNA sequencing, HOXA13

## Abstract

*In vitro* models allow for the study of developmental processes outside of the embryo. To gain access to the cells mediating digit and joint development, we identified a unique property of undifferentiated mesenchyme isolated from the distal early autopod to autonomously re-assemble forming multiple autopod structures including: digits, interdigital tissues, joints, muscles and tendons. Single-cell transcriptomic analysis of these developing structures revealed distinct cell clusters that express canonical markers of distal limb development including: *Col2a1*, *Col10a1*, and *Sp7* (phalanx formation), *Thbs2* and *Col1a1* (perichondrium), *Gdf5*, *Wnt5a*, and *Jun* (joint interzone), *Aldh1a2* and *Msx1* (interdigital tissues), *Myod1* (muscle progenitors), *Prg4* (articular perichondrium/articular cartilage), and *Scx* and *Tnmd* (tenocytes/tendons). Analysis of the gene expression patterns for these signature genes indicates that developmental timing and tissue-specific localization were also recapitulated in a manner similar to the initiation and maturation of the developing murine autopod. Finally, the *in vitro* digit system also recapitulates congenital malformations associated with genetic mutations as *in vitro* cultures of *Hoxa13* mutant mesenchyme produced defects present in *Hoxa13* mutant autopods including digit fusions, reduced phalangeal segment numbers, and poor mesenchymal condensation. These findings demonstrate the robustness of the *in vitro* digit system to recapitulate digit and joint development. As an *in vitro* model of murine digit and joint development, this innovative system will provide access to the developing limb tissues facilitating studies to discern how digit and articular joint formation is initiated and how undifferentiated mesenchyme is patterned to establish individual digit morphologies. The *in vitro* digit system also provides a platform to rapidly evaluate treatments aimed at stimulating the repair or regeneration of mammalian digits impacted by congenital malformation, injury, or disease.

## 1 Introduction

Congenital malformations of the hand or foot affect approximately one to two children/1,000 births in the United States annually (Linder et al., 2009; Goldfarb et al., 2015; Goldfarb et al., 2017; Jain et al., 2020). Despite affecting >8,000 children/year, the cellular and molecular mechanisms involved in digit and joint formation are not well understood. A significant challenge to advance our understanding of these mechanisms is the inability to directly probe mammalian cell populations as they participate in the formation of digit/joint structures. To address this challenge, Solursh and colleagues developed the micromass assay, an *in vitro* approach that identified the capacity of cultured limb mesenchyme to recapitulate endochondral skeletal development (Ahrens et al., 1977; Solursh et al., 1978; Paulsen and Solursh, 1988; Daniels et al., 1996). While micromass assays have advanced our understanding of cellular and molecular processes mediating mesenchymal cell proliferation and condensation, apoptosis, and matrix mineralization necessary for endochondral skeletal development, this approach has not provided insight into how limb mesenchyme is instructed to form additional musculoskeletal components of the hand and foot (Oberlender and Tuan, 1994; Mello and Tuan, 1999; DeLise et al., 2000; Delise and Tuan, 2002; Daumer et al., 2004; Zhang et al., 2004; Mello and Tuan, 2006; Handschel et al., 2007; Pirosa et al., 2019).

To address this knowledge gap, we expanded our initial finding that cells expressing *Hoxa13* are competent to form digit-like structures *in vitro* and examined the full developmental potential of distal limb bud mesenchyme (Stadler et al., 2001). Remarkably, when placed in culture, distal limb cells exhibit robust recapitulation of hand/foot development producing multiple musculoskeletal tissues/structures beyond the digit endochondral ossification including: interdigital tissues, joint interzones, tendons, fetal muscle, and perichondrium. Access provided to multiple mammalian hand/foot tissues as they develop in the *in vitro* digit in a dish system (DID) represents a significant breakthrough in our ability to probe and functionally characterize the molecular programs controlling mammalian limb development using high-resolution single-cell transcriptomics and hybridization chain reaction RNA fluorescence *in situ* hybridization (HCR RNA-FISH).

The DID system also appears competent to model congenital defects caused by loss of gene function, as digit structures formed using *Hoxa13* homozygous mutant cells reproduce defects in mesenchymal condensation and digit formation reported in *Hoxa13* mutant embryos (Fromental-Ramain et al., 1996; Stadler et al., 2001; Perez et al., 2010; Bastida et al., 2020). The combined assessment of control and mutant DID cultures provides a unique resource to model congenital malformations and to rapidly evaluate therapies aimed at correcting congenital malformations or to stimulate the regeneration of mammalian digits impacted by injury or disease.

Using three developmental time points, we present a comprehensive analysis of the DID system examining the transcriptomes present in >70,000 cells as they participate in the formation of hand/foot musculoskeletal tissues at single cell resolution. This analysis provides new insights into the heterogeneity of progenitor cell types present in undifferentiated distal limb mesenchyme as well as a confirmation that the DID system closely models single-cell transcriptomic studies of embryonic limbs including stage-, tissue-, and cell-type-specific expression of developmental genes (Desanlis et al., 2020; Kelly et al., 2020). Based on our transcriptomic analysis of DID cultures, we identify six distinct autopod developmental trajectories recapitulated by the DID system: endochondral cartilage and bone, perichondrium, interdigital tissues, joints, fetal muscle, and tendons. Finally, limb developmental gene expression patterns were also recapitulated by the tissues/structures forming in the DID system, providing additional confirmation that hand/foot development is modeled by this system.

## 2 Materials and methods

### 2.1 Mice

All mice were housed in a specific pathogen-free facility at the Portland Shriners Hospital. Embryos were obtained from timed matings of CD-1 mice (Charles River Labs), or from timed matings of Hoxa13^GFP^ mice maintained on a C57BL/6 and CD-1 mixed genetic background as described (Stadler et al., 2001). Estimated embryonic gestational age was determined by timed matings using the detection of a vaginal plug to establish embryonic day (E) 0.5 as described (Mader et al., 2009). Genotyping of Hoxa13^GFP^ embryos was accomplished using PCR and yolk-sac DNA as described (Stadler et al., 2001; Morgan et al., 2003).

### 2.2 Digits in a dish (DID) cultures

**FIGURE 1:**
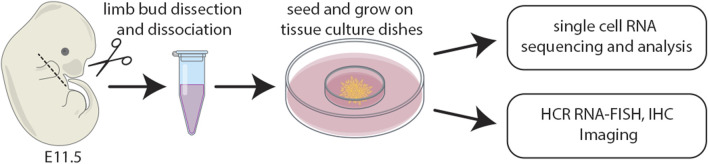
Schematic of the experimental design whereby distal limb mesenchyme was dissected, dissociated, and single cells captured. The distal most third of forelimb buds were micro-dissected and pooled from E11.5 mouse embryos. Cell suspensions were inoculated onto 60 mm tissue culture dishes and subsequently processed for either single cell RNA sequencing, HCR RNA-FISH, or IHC.

Staged limb buds (Wanek et al., 1989) from wildtype embryos at E11.5 were collected in 4°C Ca^2+^- and Mg^2+^-free phosphate-buffered saline (PBS; Gibco/BRL). Distal forelimb mesenchyme was micro-dissected using fine tungsten needles and scissors and a Leica MZ12 stereoscope. Dissected embryonic limb tissues were pooled into a 2 mL microcentrifuge tube and dissociated at 37°C for 13 minutes in Ca^2+^- and Mg^2+^-free PBS (Gibco/BRL) containing 0.1% trypsin and 0.1% collagenase (Type IV) as described (Owens and Solursh, 1982), with a vigorous flick of the microcentrifuge tube after the first 5 minutes. After the 13-min digestion, 1 mL of Dulbecco’s MEM media containing 10% FBS supplemented with non-essential amino acids, 50 U/mL penicillin and 25 μg/mL streptomycin was added to the digest with a wide bore pipette, followed by gentle pipetting 100 times with a sterile transfer pipette. The limb bud cell suspension was then passed through sterile 74 μm polyester mesh (Costar Netwells). The flow-through was centrifuged at a relative centrifugal force (RCF) of 180 g for 5 minutes in a sterile 2 mL microcentrifuge tube. The supernatant was removed and 1 mL of Dulbecco’s MEM media containing 10% FBS supplemented with non-essential amino acids, 50 U/mL penicillin and 25 μg/mL streptomycin was added to the pellet by gently trickling down the side of the 2 mL microcentrifuge tube. The tube was gently agitated to dissociate the cell pellet. The cell suspension was centrifuged for a second time at an RCF of 180 g for 5 minutes. The supernatant was removed, leaving 100 μL of supernatant in the tube which was used to dissociate the cell pellet. The cell suspension was counted on a hemocytometer and diluted to a final concentration of 2 × 10^7^cells/mL with Dulbecco’s MEM media containing 10% FBS supplemented with non-essential amino acids, 50 U/mL penicillin and 25 μg/mL streptomycin. Cell suspensions were inoculated onto 60 mm Falcon tissue culture dishes as described (Ahrens et al., 1977) and placed in a 37°C incubator with a 5% CO_2_ atmosphere for 1 h to allow for cell attachment. After 1 h, the dishes were gently filled with a max volume of media which was changed daily. Cultures were subsequently processed for either single cell RNA sequencing, HCR RNA-FISH, or immunohistochemistry (IHC) (Figure 1).

### 2.3 Single-cell isolation, capture, library construction, and sequencing

Two independent DID cultures were processed as separate replicates for scRNAseq analysis of Day 2, 7 and 10 time points following the cell dissociation and library preparation protocols as described by the manufacturer (10X Genomics). Dissociated cells were centrifuged at an RCF of 180 g for 5 minutes. Following centrifugation, the cells were washed in PBS containing 0.4% bovine serum albumin and strained through a 75 µm nylon mesh (Costar Netwells). After straining, cells were counted and adjusted to a concentration of 1,000 cells/μL for the single-cell RNAseq pipeline.

Single cells from each replicate were isolated using the 10x Genomics Chromium platform at the OHSU Integrated Genomics Laboratory. Replicate DID culture libraries were made with the 10x Genomics 3′Gene Expression, GEX V3.1 chromium kit. All libraries were sequenced at the OHSU Integrated Genomics Laboratory on the Illumina NovaSeq 6,000 sequencing system.

The following number of cells were used to produce the libraries for replicate scRNAseq analysis: Day 2, replicate A (11,518 cells, 68,572 average reads per cell), Day 2, replicate B (20,188 cells, 39,112 average reads per cell), Day 7, replicate A (16,784 cells, 58,369 average reads per cell), Day 7, replicate B (18,049 cells, 52,673 average reads per cell), Day 10, replicate A (12,313 cells, 77,728 average reads per cell), and Day 10, replicate B (12,363 cells, 73,518 average reads per cell). After quality control and filtering, 9,448, 16,485, 13,486, 12,346, 10,385, and 9,949 estimated number of high-quality cells remained in the samples, respectively (Supplementary Table S1) (Butler et al., 2018; Stuart et al., 2019). Unbiased cell clustering based on gene expression was performed on these remaining high-quality cells. (Butler et al., 2018; Stuart et al., 2019).

### 2.4 Single-cell clustering and differential expression analysis

The raw scRNAseq data was processed following the 10X Genomics pipeline with Cell Ranger (version 6.1.2) software to remove low-quality reads (Zheng et al., 2017). The remaining reads were aligned to the GRCm38 murine reference genome using STAR, and gene count matrices were generated as described (Du et al., 2020). Gene count matrices were imported into the R package Seurat (version 4.1.0) for all downstream analyses as described (Stuart et al., 2019). In Seurat, additional quality filtering was performed based on the percentage of mitochondrial reads, number of unique feature counts, number of molecules detected in each cell, and percentage of ribosomal reads. Expression matrices were normalized and stabilized for technical noise variance using the SCTransform function in Seurat as described (Hafemeister and Satija, 2019). Dimension numbers used for PCA reductions were selected based on the Seurat elbow plot. Clustree plots were used to help select the resolution parameter as described (Zappia and Oshlack, 2018). Clusters were determined using the FindNeighbors and FindClusters functions implemented in Seurat, which uses a graph-based clustering approach. Uniform Manifold Approximation and Projection (UMAP) was used for dimensionality reduction. Two independent replicates were analyzed for each time point. Integration of the two replicates was performed using the PrepSCTIntegration, FindIntegrationAnchors, and IntegrateData functions, with setting the features to integrate to include all genes. In the integrated data, we regressed out the difference between the cell cycle G2M and S phase scores during the SCTransform function step. The differential expression analysis was run using the FindAllMarkers function in Seurat using the Wilcoxon rank sum test with a minimum 0.25 log fold change between clusters (logfc.threshold = 0.25) and expressed in at least 25% of cells from the cluster (min.pct = 0.25). Cell-type-specific clusters were defined by the expression of signature factors for each cell type. Visualization of candidate gene expression was made using the VlnPlot, FeaturePlot and DotPlot functions in Seurat. Parameters for the individual libraries included the percentage of mitochondrial reads (%Mito), upper limit to the number of unique feature counts (FeatUp), lower limit to the number of unique feature counts (FeatLow), percentage of ribosomal reads (%Ribo), PCA dimensions (PCAdims), and resolution (Res). In addition, a lower limit to RNA read counts (UMI counts) was set to filter signals that were overcalled as cells by Cell Ranger (UMIcount). Parameters were set as the following for the individual libraries: Day 2, replicate A (%Mito = 25, FeatUp = 7,500, FeatLow = 200, %Ribo = 35, PCAdims = 35, Res = 0.7, UMIcount = 800). Day 2, replicate B (%Mito = 25, FeatUp = 7,500, FeatLow = 200, %Ribo = 35, PCAdims = 35, Res = 0.7, UMIcount = 1,000). Day 7, replicate A (%Mito = 30, FeatUp = 7,500, FeatLow = 200, %Ribo = 40, PCAdims = 35, Res = 0.5, UMIcount = 1,000). Day 7, replicate B (%Mito = 30, FeatUp = 7,500, FeatLow = 200, %Ribo = 40, PCAdims = 35, Res = 0.5, UMIcount = 1,000). Day 10, replicate A (%Mito = 40, FeatUp = 7,500, FeatLow = 200, %Ribo = 40, PCAdims = 35, Res = 0.7, UMIcount = 800). Day 10, replicate B (%Mito = 40, FeatUp = 7,500, FeatLow = 200, %Ribo = 35, PCAdims = 35, Res = 0.5, UMIcount = 800) (Supplementary Table S1).

### 2.5 FACS enrichment of *Hoxa13* ^*GFP*^-expressing cells

Distal mesenchyme expressing *Hoxa13* ^*GFP*^ was collected from E11.5 heterozygous- and homozygous *Hoxa13* ^*GFP*^ mutant embryos as described (Stadler et al., 2001). The dissected mesenchyme was digested and enriched for the *Hoxa13* ^*GFP*^-expressing cells by fluorescence activated cell sorting using a BD LSR-II (BD Biosciences) provided by the OHSU Flow Cytometry Shared Resource Facility. Heterozygous- or homozygous mutant FACS-enriched samples were used for seeding individual DID cultures as described (Stadler et al., 2001). Analysis of *Hoxa13* ^*GFP*^-expressing cells contributing to *in vitro* digit formation was performed using a Zeiss LSM 700 confocal microscope. Image processing was done using Adobe Photoshop (Version 24.1.0).

### 2.6 Antibodies and immunohistochemistry

DID cultures were washed twice (5 minutes/wash) in PBS and fixed in 4% paraformaldehyde for 10 min at room temperature. After fixation, the samples were washed three times using room temperature PBS (5 minutes/wash) and incubated for 1 hour with PBSTMD (PBS, 2% powdered milk, 0.5% Triton X-100, 2% whole donkey serum). After 1 hour, fresh PBSTMD containing a 1:250 dilution of antibodies specific for Tenomodulin (TNMD) (Abcam: AB203676) or SOX9 (Novus Biologicals: AF3075-SP) was added to the samples which were incubated at 4°C overnight. The next day, primary antibody solutions were removed and the samples were washed three times (5 minutes/wash) in room temperature PBSTMD. After the final wash, the samples were incubated overnight at 4°C in PBSTMD containing a 1:400 dilution of a donkey anti-goat- or donkey anti-rabbit secondary antibody conjugated with Alexa 488 (Jackson ImmunoResearch Laboratories:705-545-003) or Alexa 647 (Jackson ImmunoResearch Laboratories: 711-605-152). The next day, samples were washed three times in PBS (5 minutes/wash), followed by staining with DAPI/PBS for 5 minutes (ThermoFisher: 62248). After DAPI staining, the samples were washed twice in room temperature PBS (5 minutes/wash). Samples were imaged for immunolocalization of SOX9 or TNMD using a Zeiss LSM 700 confocal microscope as described (Morgan et al., 2003). Image processing was done using Adobe Photoshop (Version 24.1.0).

### 2.7 Whole mount *in situ* hybridization analysis of *Hoxa13* and *tnmd* expression

A 310 base-pair region corresponding to nucleotides 650–960 of the murine *Hoxa13* cDNA sequence (NM_008264) was amplified from C57BL/6 genomic DNA by PCR using the following primers: A13_Exon1 F: 5′-TAC​CCG​TGC​GCC​CGC​AT-3′ and A13_Exon1′R: 5′-CCG​TTC​CAG​CCG​TTG​GG-3’. This region was selected for *Hoxa13* expression analysis due to its presence in wild-type- and *Hoxa13* ^GFP^-mutant alleles. The amplified DNA region was cloned into a t-tailed vector containing RNA polymerase T3 and T7 promoters. *Hoxa13* riboprobe synthesis, embryo preparation, riboprobe hybridization and colorimetric detection were performed as described (Manley and Capecchi, 1995). Embryos were photographed using a Leica MZFL12 stereoscope fitted with a Canon EOS 40D digital camera. Image processing was done using Adobe Photoshop (Version 24.1.0).

Whole-mount *in situ* hybridization analysis of *Tnmd* expression was performed as described (Shukunami et al., 2018). Briefly, embryos were fixed in 4% PFA/PBS overnight and dehydrated with methanol. After rehydration and proteinase K treatment, embryos were hybridized with DIG-labelled probes at 70°C overnight. After washing, the hybridized probes were detected by using alkaline phosphatase conjugated anti-DIG antibody (Roche) and BM purple (Roche).

### 2.8 Hybridization chain reaction RNA fluorescence *in situ* hybridization (HCR RNA-FISH)

We adapted the HCR RNA-FISH protocol for fixed frozen sections on slides for use with the DID cultures (Molecular Instruments). Gene-specific HCR probe sets, buffers, and amplification hairpins were purchased from Molecular Instruments (Los Angeles, CA) (Table 1). DID cultures used for HCR RNA-FISH were grown on 60 mm × 15 mm Center-Well Organ Culture Dishes (Corning: Falcon Ref 353037). Media was removed and rinsed twice with phosphate buffered saline (PBS) on ice. Samples were fixed for 15 minutes with fresh 4% paraformaldehyde in PBS (PFA/PBS) at 4°C and then rinsed twice with PBS for 5 minutes. After fixation, cultures were digested with proteinase K (7 μg/mL in PBS) for 10 minutes at 37°C. Digested cultures were rinsed twice with PBT (PBS +0.1% Tween 20) for 5 minutes, and re-fixed using 4% PFA/PBS for 5 minutes. The re-fixed samples were rinsed twice with PBT for 5 min. A prehybridization step using probe hybridization buffer (Molecular Instruments) heated to 37°C was added to the samples which were then incubated in a humidified chamber for 10 minutes inside a 37°C oven. After prehybridization, a comparable volume of hybridization buffer containing the gene-specific probes (0.4 pmol/100 µL hybridization buffer) was added to the samples as recommended by the manufacturer (Molecular Instruments). Samples were incubated overnight in humidified chamber in a 37°C oven. The next day, the probe solution was removed and the plates were washed with 75% probe wash buffer/25% 5X SSCT for 15 minutes at 37°C followed by a wash using 50% probe/50% 5X SSCT for 15 minutes, 25% probe wash buffer/75% 5X SSCT for 15 minutes, 100% 5X SSCT for 15 minutes, and then 100% 5X SSCT for 5 minutes at room temperature as recommended by the manufacturer (Molecular Instruments). Samples were covered with amplification buffer (Molecular Instruments) and placed in a humidified chamber for 30 minutes at room temperature. After pre-amplification, the buffer was removed and snap-cooled amplification hairpins (6 pmol/hairpin) were added to the samples as recommended by the manufacturer (Molecular Instruments). Samples were placed in humidified chambers wrapped with aluminum foil and incubated overnight in a dark drawer at room temperature. The following day, the hairpin/amplification solution was removed and the samples were washed two times for 30 minutes in 5X SSCT at room temperature followed by a single 5-min wash in 5X SSCT. Cultures were then stained for 5 minutes with a DAPI solution as described by the manufacturer (ThermoFisher: 62248). After DAPI staining, the cultures were rinsed twice with PBS and imaged in PBS with a Zeiss LSM-700 confocal microscope. Image processing was done using Adobe Photoshop (Version 24.1.0).

**TABLE 1:** HCR RNA-FISH probe sets. Below is the list of probes used for HCR RNA-FISH in this study, including the HCR amplifier paired with each probe and the probe set size.

Gene	Accession number	Amplifier	Probe set size
*Aldh1a2*	BC_075704.1	B3	20
*Col2a1*	NM_031163.3	B3	20
*Gdf5*	NM_008109.3	B4	20
*Hoxa13*	NM_008264	B2	12
*Myod1*	NM_010866.2	B4	20
*Prg4*	NM_021400.3	B4	20
*Thbs2*	NM_011581.3	B5	20

## 3 Results

### 3.1 Development of the digits in a dish system

**FIGURE 2:**
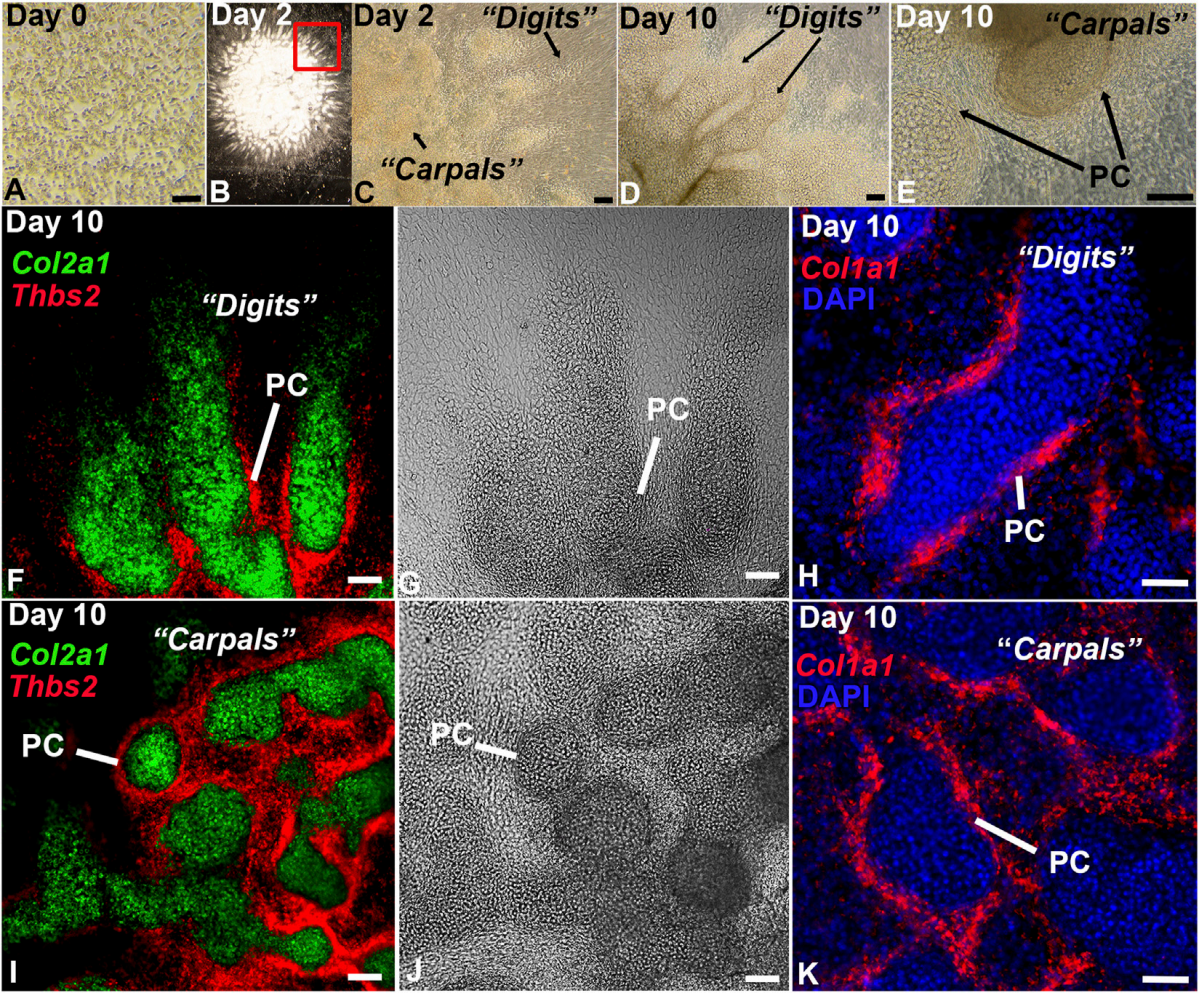
*In vitro* culture of distal limb mesenchyme recapitulates digit and carpal element formation. **(A)** Brightfield image of the disassociated E11.5 distal forelimb mesenchyme at Day 0. **(B)** Low magnification image of an entire DID culture at Day 2 showing re-assembly and development of the dissociated mesenchyme into digit-like projections. **(C)** Higher magnification image of the boxed region in panel **(B)**. Arrows denote digit- and carpal-like elements. **(D, E)** Analysis of Day 10 DID cultures revealed well-formed digits and carpal elements producing a visible perichondrium (PC). **(F–K)** HCR RNA-FISH analysis of gene expression in Day 2 DIDs reveal robust expression of *Col2a1* in the central condensations and *Col1a1* and *Thbs2* localizing to the tissue surrounding these structures, consistent with the formation of a perichondrium (PC). Bars = 50 µm.

While high density micromass culture of limb bud mesenchyme typically develops into rounded endochondral nodules (Ahrens et al., 1977; Solursh et al., 1978; Carlson et al., 2015), cells disassociated from the distal limb bud exhibited a remarkable capacity to reassemble *in vitro*, producing digit-, joint-, and carpal/tarsal-like structures with a perichondrium (Figure 2). The autonomous reassembly of this cell population into digit- and carpal/tarsal-like structures indicates an expanded hand/foot developmental program is recapitulated by the DID system without the use of instructive extracellular matrices, tissue-engineering scaffolds, or exogenous growth factor applications, other than those provided by fetal bovine serum (Ahrens et al., 1977; Solursh et al., 1978; Ahrens et al., 1979; Stadler et al., 2001; Moutos et al., 2007; Huynh et al., 2018). Testing this hypothesis, we characterized the full developmental potential of distal limb mesenchyme in DID cultures using single-cell transcriptomics and HCR RNA-FISH (Figures 1, 2).

### 3.2 Distal limb mesenchyme is comprised of heterogeneous cell types that mediate initial stages of autopod development in day 2 DID cultures

To better define the cell types and developmental programs mediating *in vitro* digit formation, we analyzed the transcriptomes present in DID cultures using single-cell RNA-seq (scRNAseq). Three time points were selected to define the cell-specific transcriptomes defining hand musculoskeletal development at single cell resolution using the 10X Genomics platform. A time point of two days after seeding the DID cultures (Day 2) was selected to capture transcriptomic events mediating early stages of digit initiation. A Day 7 time point was selected to capture the transitional transcriptomes mediating digit/joint specification, and a Day 10 time point was selected to detect the transcriptomes mediating the maturation of hand/foot musculoskeletal tissues and structures. Two independent samples were analyzed by scRNAseq at each time point to assess variability between DID cultures and to ensure reproducibility of the scRNAseq results.

**FIGURE 3:**
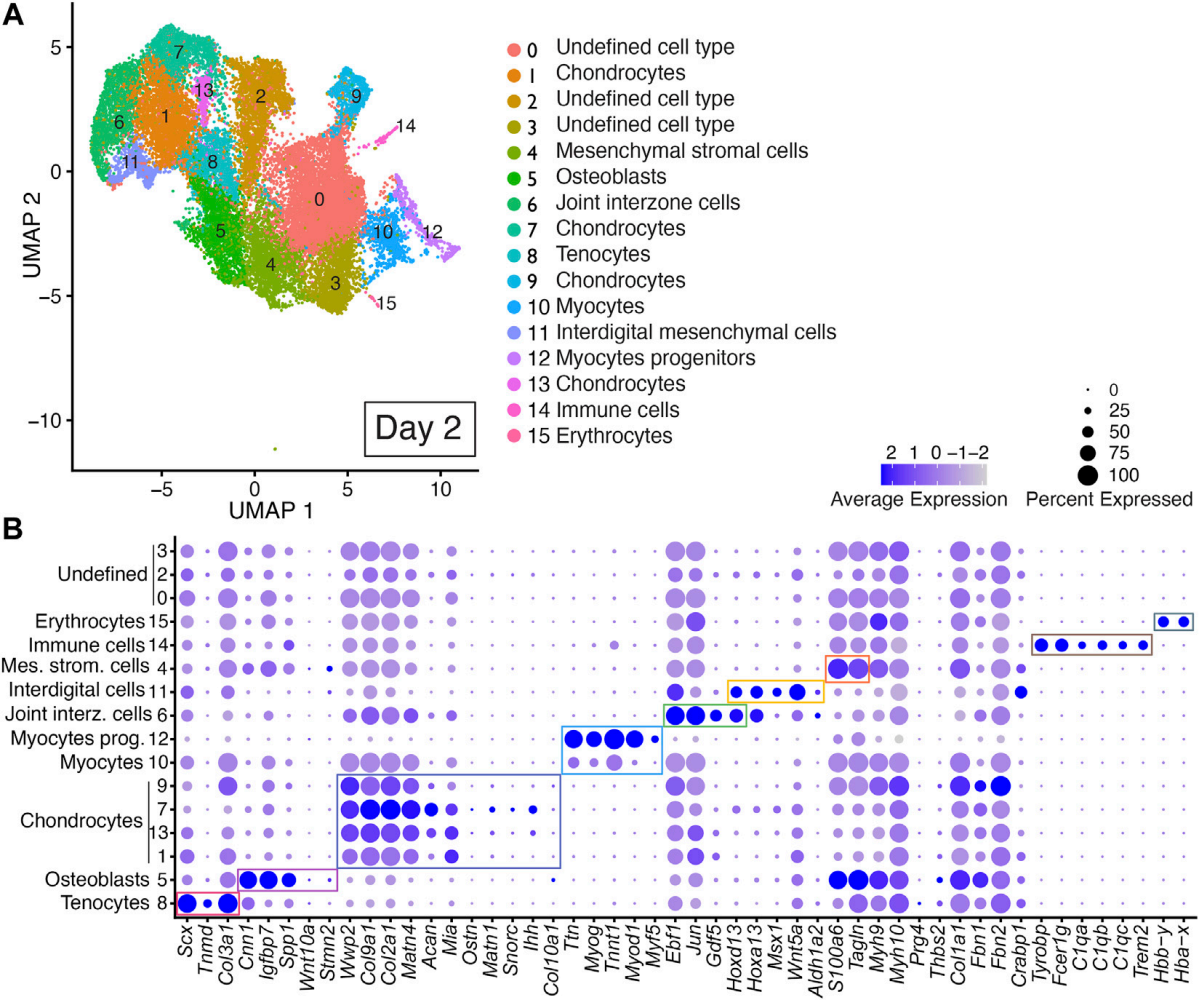
scRNAseq analysis of Day 2 *in vitro* cultures identifies multiple early autopod cell types. **(A)** UMAP (Uniform Manifold Approximation and Projection) plot of Day 2 culture scRNAseq results demonstrate 16 different clusters of identifiable cell populations. Individual cells are color coded based on cluster and marker gene annotation denoting tenocytes (Clusters 8), osteoblasts (Cluster 5), chondrocytes (Clusters 1, 7, 9, and 13), myocytes (Cluster 10), myocyte progenitors (Cluster 12), joint interzone cells (Cluster 6), interdigital mesenchymal cells (Cluster 11), mesenchymal stromal cells (Cluster 4), immune cells (Cluster 14), erythrocytes (Cluster 15), and undefined cell types (Clusters 0, 2, 3). **(B)** Dot plots highlighting expression profiles of selected marker genes per cell type used for cluster cell identification. Colored rectangles highlight the subset of markers used to identify tenocytes (red), osteoblasts (pink), chondrocytes (purple), myocytes/myocyte progenitors (blue), joint interzone (green), interdigital mesenchyme (orange), immune cell lineages (brown) and erythrocytes (gray). Dot diameter corresponds to the percentage of cells expressing the gene in each cluster. High-Low expression is denoted by Purple-Gray bar, respectively.

Unbiased clustering of the integrated dataset from Day 2 scRNAseq replicates revealed 16 discrete cell populations represented by Clusters 0–15 (Figure 3; Supplementary Table S2). Cell type identity was determined using published cell-type-specific gene expression and cell annotation and ontology applications Panglao DB and ShinyGO 0.76.2 (Franzen et al., 2019; Ge et al., 2020). Cell types assigned to Cluster 8 were annotated as tenocytes based on their expression of *Scx*, *Tnmd*, and *Col3a1* (Figure 3, Supplementary Table S2) (Shukunami et al., 2006; Shukunami et al., 2018; Qi et al., 2020). Cell types assigned to Cluster 5 were annotated as osteoblasts based on their expression of *Cnn1*, *Igfbp7*, and *Spp1* (Figure 3, Supplementary Table S2) (Moore et al., 1991; Su et al., 2013; Wu et al., 2019; Lu et al., 2020). Chondrocytes were identified by their expression of *Wwp2*, *Col9a1*, *Col2a1*, *Acan*, and *Matn4*, *Mia*, *Ostn*, *Snorc*, and *Matn1* and were assigned to Clusters 1, 7, 9, and 13 (Figure 3 and Supplementary Table S2) (Bi et al., 1999; Makihira et al., 1999; Czarny-Ratajczak et al., 2001; Moser et al., 2002; Rentsendorj et al., 2005; Domowicz et al., 2009; Groma et al., 2011; Heinonen et al., 2011; Yang et al., 2011). Cell types assigned to Clusters 10 and 12 were annotated as myocyte progenitors or myocytes based on their expression of *Ttn*, *Myog*, *Myod1*, *Myf5*, and *Tnnt1* (Figure 3; Supplementary Table S2) (Tajbakhsh et al., 1996; Beauchamp et al., 2000; Linke and Kruger, 2010; Wood et al., 2013; Ganassi et al., 2018; Li et al., 2021; Yagi et al., 2021). Cell types assigned to Cluster 6 were annotated as joint interzone cells based on their expression of *Ebf1*, *Jun*, *Hoxd13*, and *Gdf5* (Figure 3, Supplementary Table S2) (Storm and Kingsley, 1999; Kan and Tabin, 2013; Huang et al., 2016; Shwartz et al., 2016; Capellini et al., 2017; El-Magd et al., 2021). Cell types assigned to Cluster 11 were annotated as interdigital mesenchyme based on their significant expression of several interdigital tissue markers including: *Hoxa13*, *Hoxd13*, *Msx1*, *Wnt5a*, and *Aldh1a2* (Figure 3; Supplementary Figure S3).

Cells assigned to Cluster 4 were annotated as mesenchymal stromal cells based on their expression of *Tagln* and *S100a6* (Figure 3; Supplementary Table S2) (Marin-Llera and Chimal-Monroy, 2018; Luo et al., 2020). An immune cell cluster, Cluster 14, was also identified in the Day 2 cultures based on the expression of *Tyrobp*, *Fcer1g*, *C1qa*, *C1qb*, *C1qc*, and *Trem2* (Figure 3; Supplementary Table S2) (Chen et al., 2021; Liang et al., 2021; Qiu et al., 2021; Dong et al., 2022). Cells assigned to Cluster 15 were annotated as erythrocytes based on their expression of *Hbby-y* and *Hba-x* (Figure 3; Supplementary Table S2) (Holdener-Kenny and Weaver, 1986; Alhashem et al., 2011; Chatterjee et al., 2016). Cells assigned to Clusters 0, 2, and 3 could not be annotated due to a lack of enrichment of informative markers (Figure 3; Supplementary Table S2).

A comparison of the Day 2 scRNAseq datasets indicated high reproducibility of digit developmental programs in independent DID cultures with cell-type-specific gene expression consistently reproduced between replicates for the 49-member gene list selected to capture six distinct autopod developmental trajectories including: digits and carpal elements, perichondrium, interdigital tissues, joints, fetal muscle, and tendons (Figure 3, Supplementary Figures S1, S2). The contribution of cells expressing cluster-specific genes was quite similar between replicate Day 2 DID libraries indicating a reproducible developmental program is produced by the DID system with the exception of Clusters 0, 3, 4, and 10 which exhibited a higher percentage of cells contributed from replicate B (Supplementary Figure S1).

### 3.3 Distinct autopod musculoskeletal tissues and structures are present in day 7 DID cultures

To determine whether distinct musculoskeletal tissues and structures are maturing in the DID system, we next analyzed the integrated scRNAseq datasets from Day 7 replicate libraries. Unbiased cell clustering based on gene expression was performed on the high-quality cells that remained after standard quality control and read filtering was performed in Seurat (Supplementary Table S1) as described (Butler et al., 2018; Stuart et al., 2019).

**FIGURE 4:**
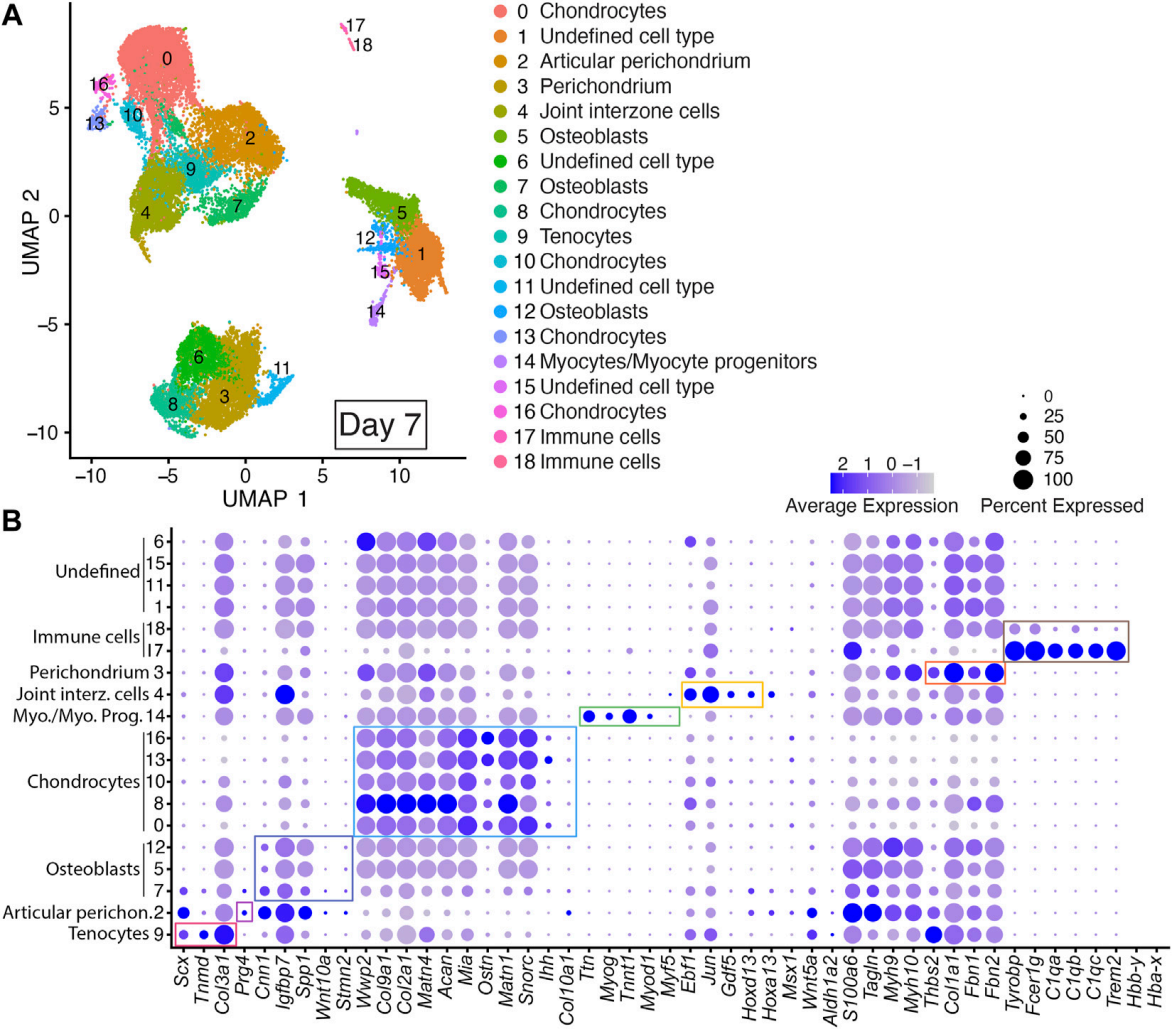
scRNAseq analysis of Day 7 *in vitro* cultures identifies multiple early autopod cell types. **(A)** UMAP (Uniform Manifold Approximation and Projection) plot of Day 7 culture scRNAseq results demonstrate 19 different clusters of identifiable cell populations. Individual cells are color coded based on cluster and marker gene annotation denoting tenocytes (Cluster 9), articular perichondrium (Cluster 2), osteoblasts (Clusters 5, 7 and 12), chondrocytes (Clusters 0, 8, 10, 13, and 16), myocytes/myocyte progenitors (Cluster 14), joint interzone cells (Cluster 4), perichondrium (Cluster 3), immune cell lineages (Clusters 17 and 18) and undefined cell types (Clusters 1, 6, 11, and 15). **(B)** Dot plots highlighting expression profiles of selected marker genes per cell type used for cluster cell identification. Colored rectangles highlight the subset of markers used to identify tenocytes (red), articular perichondrium (pink), osteoblasts (purple), chondrocytes (blue), myocytes/myocyte progenitors (green), joint interzone cells (yellow), perichondrium (orange), and immune cell lineages (brown). Dot diameter corresponds to the percentage of cells expressing the gene in each cluster. High-Low expression is denoted by Purple-Gray bar, respectively.

Integrated cell clustering analysis for the two Day 7 DID libraries revealed 19 discrete populations represented by Clusters 0–18 (Figure 4; Supplementary Table S2). Cells assigned to Cluster 9 were annotated as tenocytes based on their expression of *Scx*, *Tnmd*, and *Col3a1* (Shukunami et al., 2006; Shukunami et al., 2018; Qi et al., 2020). Cells assigned to Clusters 5, 7, and 12 were annotated as osteoblasts based on their expression of *Cnn1*, *Igfbp7*, *Spp1*, *Wnt10a*, and *Stmn2* (Figure 4; Supplementary Table S2) (Moore et al., 1991; Chiellini et al., 2008; Cawthorn et al., 2012; Su et al., 2013; Wu et al., 2019; Lu et al., 2020). Cells assigned to Clusters 0, 8, 10, 13, and 16 were annotated as chondrocytes based on their expression of multiple chondrocyte markers including: *Wwp2*, *Col9a1*, *Col2a1*, *Matn4*, *Acan*, *Mia*, *Ostn*, *Matn1*, *Snorc*, *and Ihh* (Figure 4; Supplementary Table S2) (Bi et al., 1999; Makihira et al., 1999; Czarny-Ratajczak et al., 2001; Kobayashi et al., 2002; Moser et al., 2002; Rentsendorj et al., 2005; Domowicz et al., 2009; Groma et al., 2011; Heinonen et al., 2011; Yang et al., 2011). Cells assigned to Cluster 14 were annotated as myocytes or myocyte progenitors based on their expression of *Myod1*, *Ttn*, *Myf5*, *Myog*, and *Tnnt1* (Figure 4; Supplementary Table S2) (Bi et al., 1999; Makihira et al., 1999; Czarny-Ratajczak et al., 2001; Moser et al., 2002; Rentsendorj et al., 2005; Domowicz et al., 2009; Groma et al., 2011; Heinonen et al., 2011; Yang et al., 2011).

Cells assigned to Cluster 4 were annotated as joint interzone based on their co-expression of several joint interzone markers including: *Jun*, *Gdf5*, and *Hoxd13* (Figure 4; Supplementary Table S2) (Storm and Kingsley, 1999; Kan and Tabin, 2013; Chen et al., 2016; Shwartz et al., 2016). Analysis of the Day 7 datasets revealed a novel cell type we annotated as perichondrium (Cluster 3) based on the expression of several perichondrial cell markers including: *Thbs2*, *Col1a1*, *Fbn2*, and *Fbn1* (Figure 4; Supplementary Figure S3, and Supplementary Table S2) (Reinhardt et al., 1996; Kyriakides et al., 1998; Bandyopadhyay et al., 2008; Charbonneau et al., 2010). Similarly, the Day 7 datasets also identified an articular perichondrium cluster, Cluster 2, based on the expression of *Prg4* (Figure 4; Supplementary Table S2) (Kozhemyakina et al., 2015; Zhang et al., 2021). Cells assigned to Clusters 17 and 18 were annotated as immune cell types based on their co-expression of *Tyrobp*, *Fcer1g*, *C1qa*, *C1qb*, *C1qc*, and *Trem2* (Figure 4; Supplementary Table S2) (Chen et al., 2021; Liang et al., 2021; Qiu et al., 2021; Dong et al., 2022). Cells assigned to Clusters 1, 11, and 15 could not be annotated due to a lack of enrichment of informative markers in the Day 7 scRNAseq datasets (Figure 4; Supplementary Table S2).

A comparison of the Day 7 scRNAseq datasets confirmed high reproducibility of the digit developmental programs at this time point with a well-balanced contribution of cells expressing canonical markers of digit, perichondrium, interdigital tissues, joint interzone, fetal muscle, and tendons in each replicate (Figure 4, Supplementary Figures S1, S3). Cell-type-specific expression of genes was also reproduced in the Day 7 replicates including *Prg4* in the articular perichondrium (Cluster 2), *Scx*, *Col3a1*, and *Tnmd* in tenocytes (Cluster 9), *Jun*, *Ebf1*, *Gdf5*, *Hoxa13*, *Hoxd13* in joint interzone (Cluster 4), and *Thbs2*, *Col1a1*, *Fbn1*, and *Fbn2* in perichondrium (Cluster 3) (Figure 4, Supplementary Figures S1, S3).

### 3.4 Musculoskeletal tissue maturation is recapitulated in day 10 DID cultures

**FIGURE 5:**
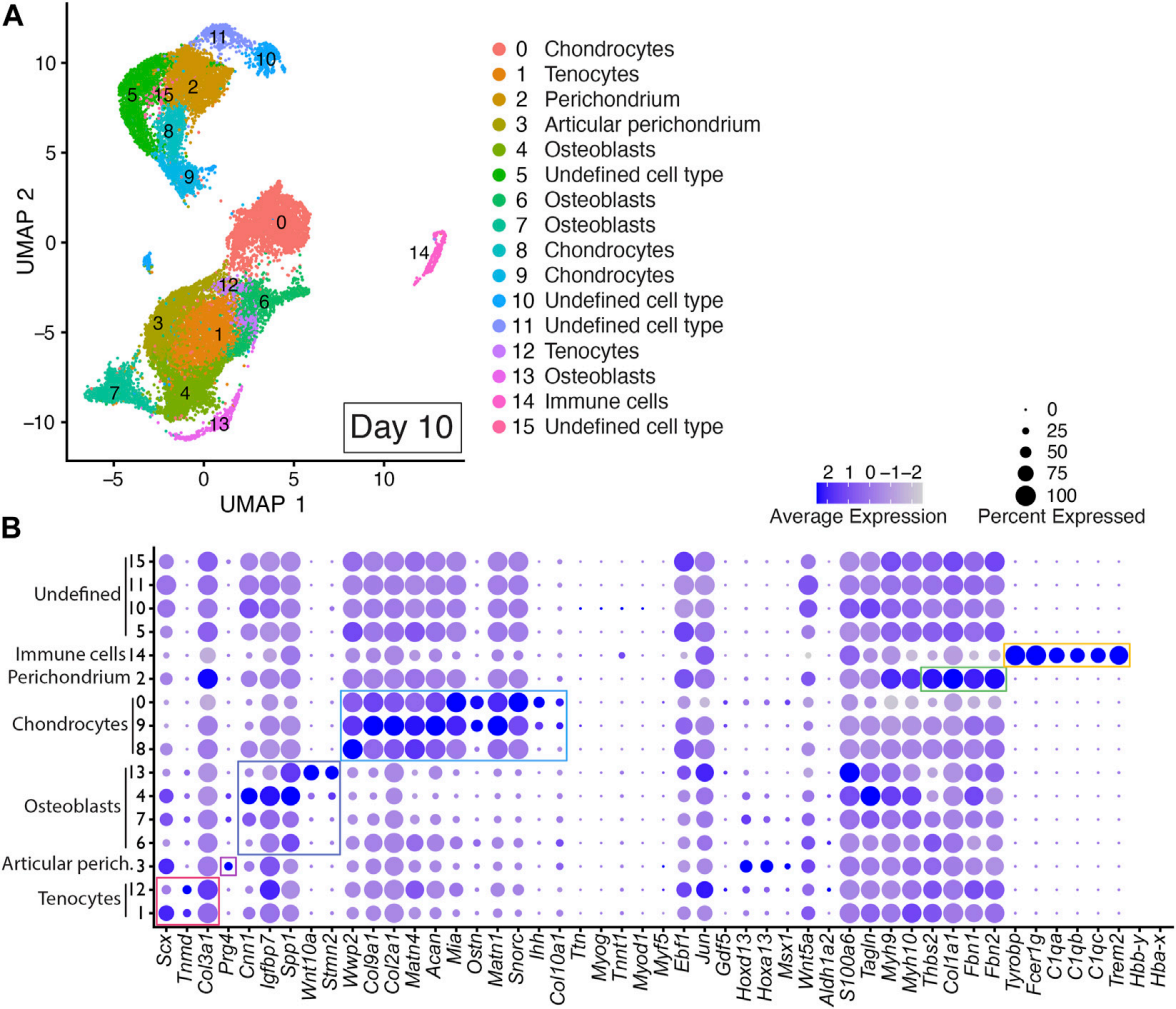
scRNAseq analysis of Day 10 *in vitro* cultures identifies multiple mature autopod cell types. **(A)** UMAP (Uniform Manifold Approximation and Projection) plot of Day 10 culture scRNAseq results demonstrate 16 different clusters of identifiable cell populations. Individual cells are color coded based on cluster and marker gene annotation denoting tenocytes (Clusters 1 and 12), articular perichondrium (Cluster 3), osteoblasts (Clusters 4, 6, 7, and 13), chondrocytes (Clusters 0, 8, and 9), articular perichondrium (Cluster 3), perichondrium (Cluster 2), immune cells (Cluster 14), and undefined cell type (Clusters 5, 10, 11, 15). **(B)** Dot plots highlighting expression profiles of selected marker genes per cell type used for cluster cell identification. Colored rectangles highlight the subset of markers used to identify tenocytes (red), articular perichondrium (pink), osteoblasts (purple), chondrocytes (blue), perichondrium (green), and immune cells (yellow). Dot diameter corresponds to the percentage of cells expressing the gene in each cluster. High-Low expression is denoted by Purple-Gray bar, respectively.

Integrated cell clustering analysis of the two Day 10 DID libraries revealed 16 discrete populations represented by Clusters 0–15 (Figure 5 and Supplementary Table S2). Cells assigned to Clusters 1 and 12 were identified as tenocyte clusters based on their expression of *Tnmd*, *Scx*, and *Col3a1* (Figure 5; Supplementary Table S2) (Shukunami et al., 2006; Shukunami et al., 2018; Qi et al., 2020). Cells assigned to Clusters 0, 8, and 9 exhibited significant enrichment of *Wwp2*, *Col9a1*, *Col2a1*, *Acan*, *Matn1*, *Matn4*, *Mia*, *Ostn*, *Snorc*, *Col10a1*, and *Ihh*, indicating a chondrocyte/growth plate chondrocyte lineage (Figure 5, Supplementary Table S2) (Apte et al., 1992; Bi et al., 1999; Czarny-Ratajczak et al., 2001; Moffatt et al., 2007; Heinonen et al., 2011; Yang et al., 2011; Schmid and Bosserhoff, 2014). Cells assigned to Clusters 4, 6, 7, and 13 were annotated as osteoblasts based on significant enrichment of *Spp1*, *Wnt10a*, *Igfbp7*, *Cnn1*, and *Stmn2* (Figure 5, Supplementary Table S2) (Moore et al., 1991; Chiellini et al., 2008; Platt and El-Sohemy, 2009; Cawthorn et al., 2012; Su et al., 2013; Ozeki et al., 2016; Wu et al., 2019; Lu et al., 2020).

The presence of *Prg4*-expressing cells in Cluster 3 in Day 10 datasets indicates maturation of the digit and carpal-like structures as this marker is normally detected in postnatal articular tissues including articular perichondrium, articular cartilage and joint capsule (Kozhemyakina et al., 2015; Zhang et al., 2021). Based on this expression, Cluster 3 was annotated as articular perichondrium (Figure 5; Supplementary Table S2). Cells assigned to Cluster 2 in the Day 10 datasets were annotated as perichondrium based on the robust expression of *Thbs2*, *Col1a1*, *Fbn1*, and *Fbn2* (Figure 5; Supplementary Table S2) (Claassen et al., 1995; Reinhardt et al., 1996; Kyriakides et al., 1998; Bandyopadhyay et al., 2008; Charbonneau et al., 2010). A comparison of *Thbs2*, *Col1a1*, *Fbn1*, and *Fbn2* expression in Day 7 and Day 10 datasets revealed increased expression for every perichondrium marker at the Day 10 time point, indicating a progressive maturation of this tissue in the DID cultures (Figures 4, 5; Supplementary Table S2). Cells assigned to Cluster 14 were annotated as immune cells based on significant enrichment of *Tyrobp*, *Fcer1g*, *C1qa*, *C1qb*, *C1qc*, *Trem2* (Figure 5; Supplementary Table S2) (Chen et al., 2021; Liang et al., 2021; Dong et al., 2022). Cells assigned to Clusters 5,10, 11, and 15 could not be annotated in the Day 10 datasets due to a lack of enrichment of informative markers (Figure 5; Supplementary Table S2).

Analysis of replicate Day 10 DID datasets also revealed high reproducibility of the digit developmental programs in independent DID cultures with cell-type-specific gene expression consistently represented between replicates for the 49-member developmental gene list used to annotate cells contributing to digits and carpal elements, digit and carpal perichondrium, interdigital tissues, joints, fetal muscle, and tendons (Figure 5, Supplementary Figures S1, S4). The contribution of cells assigned to individual clusters continued to be well-balanced between Day 10 replicate datasets (Supplementary Figure S1). Cell-type-specific expression of genes indicative of musculoskeletal tissue maturation was also reproduced in the Day 10 replicates including *Prg4* in the articular perichondrium (Cluster 3), *Col10a1* in hypertrophic chondrocytes (Clusters 0, 9), and *Thbs2*, *Col1a1*, *Fbn1*, and *Fbn2* in perichondrium (Cluster 2) (Figure 5, Supplementary Figures S1, S4).

### 3.5 Recapitulation of *Epha7* expression during DID culture mesenchymal condensation

We previously established a role for *Epha7* as a mediator of limb mesenchymal condensation (Stadler et al., 2001). Based on this finding, we hypothesized that condensing mesenchyme present in Day 2 DID cultures (Figures 2, 7) would also express *Epha7*. Analysis of the scRNAseq datasets confirmed this hypothesis which detected *Epha7* as a significantly expressed marker in Day 2 cultures for chondrocyte clusters (Clusters 1 and 13) (Figure 3, Supplementary Table S2). Elevated levels of *Epha7* expression were also present in the tenocyte cluster (Cluster 8) in Day 2 cultures (Figure 3; Supplementary Figure S5).

### 3.6 DID cultures contain endothelial and angioblast progenitors

Analysis of endothelial marker expression in the Day 2, 7, and 10 scRNAseq datasets revealed significant differential expression of several endothelial progenitor markers including *Cd34* (Day 10: Cluster 13), *Kdr* (Day 7: Cluster 17 and Day 10: Cluster 14), and *Icam1* (Day 7: Clusters 9, 17; and Day 10: Cluster 12) (Supplementary Figure S6 and Supplementary Table S2) (Fadini et al., 2006; Sidney et al., 2014; Patel et al., 2016; Patel et al., 2017). Significant differential expression was also detected for *Vcam1* in the DID cultures at Day 2 (Clusters 1 and 11), Day 7 (Clusters 9 and 17), and Day 10 (Clusters 1, 4, 12, and 13). *Pecam1* (*Cd31*) and *Tek* (*Tie2*) were not significantly represented in any cluster cell type in the Day 2, 7, and 10 scRNAseq datasets, suggesting that maturation of limb bud vascular network has not occurred in the DID cultures (Supplementary Figure S6 and Supplementary Table S2) (Patel et al., 2016; Patel et al., 2017).

We next examined *Vegfa* which is expressed in early limb buds during mesenchymal condensation to stimulate the formation of the initial vascular network and later in maturing limbs in hypertrophic chondrocytes to direct perichondrial angiogenesis and vascularization of maturing limb skeletal tissues (Petersen et al., 2002; Zelzer et al., 2002; Eshkar-Oren et al., 2009; Takimoto et al., 2009). Analysis of the scRNAseq datasets revealed significant enrichment of *Vegfa* at Day 2 (Cluster 11), Day 7 (Clusters 4 and 8) and Day 10 (Clusters 6 and 9) (Supplementary Figure S7 and Supplementary Table S2). Significant enrichment of the hypertrophic chondrocyte marker, *Col10a1*, was also seen in Day 10 cultures in the same clusters (Clusters 6 and 9) as *Vegfa* (Supplementary Figure S7 and Supplementary Table S2). This result is consistent with the previously identified role for *Vegfa* in hypertrophic chondrocytes during skeletal tissue vascularization (Takimoto et al., 2009). Finally, the *Vegfa* receptor, *Flt1*, was also significantly expressed in Day 2 (Cluster 5) and Day 10 (Cluste 4) DID cultures (Supplementary Figure S7 and Supplementary Table S2).

### 3.7 Expression of dorsal-ventral and anterior-posterior limb markers in DID cultures

The dorsal limb bud marker, *Wnt7a*, had no significant enrichment in any cell clusters with low or undetectable levels present the Day 2, 7, and 10 cultures (Supplementary Figure S8, Supplementary Table S2) (Parr and McMahon, 1995). In contrast, significant differential expression of the dorsal mesenchymal marker, *Lmx1b*, was detected in Cluster 8 in the Day 2 scRNAseq dataset which was annotated as the tenocyte cell cluster (Figure 3; Supplementary Figure S8, Supplementary Table S2). This finding, in conjunction with the lack of *Lmx1b* enrichment in the Day 7 and 10 DID clusters, is consistent with previous studies that identified *Lmx1b* expression in dorsal limb tendons between E13.5 and E15.5 with little or no expression in E16.5 limbs, (Dreyer et al., 2004; Qiu et al., 2009). The ventralizing factor, *En1,* also exhibited no significant enrichment in any cell clusters at any time point with low expression being detected in a small number of cells in the Day 2, 7, and 10 datasets (Supplementary Figure S8, Supplementary Table S2) (Loomis et al., 1996).

Anterior-posterior marker expression was also less established in the DID cultures with a notable absence of *Shh*-expressing cells in the Day 2, 7 and 10 scRNAseq datasets (data not shown). This result was likely caused by the omission of the *Shh*-expressing region in the dissected E11.5 limb bud tissue used to establish the DID cultures or by DID cultures modeling normal developmental decreases in *Shh* expression which occurs in limb buds older than E11.5 (Scherz et al., 2004; McGlinn and Tabin, 2006; Zhu et al., 2008). In contrast, an anterior marker of limb bud development, *Asb4*, was significantly enriched in Clusters 1, 7, and 13 in the Day 2 cultures as well as in cell numbers below the significant marker threshold in Clusters 2, 5, 6, 8, and 11 (Figure 3, Supplementary Figure S9, and Supplementary Table S2) (Yokoyama et al., 2017).

### 3.8 Limb developmental gene expression patterns are recapitulated by the DID system

**FIGURE 6:**
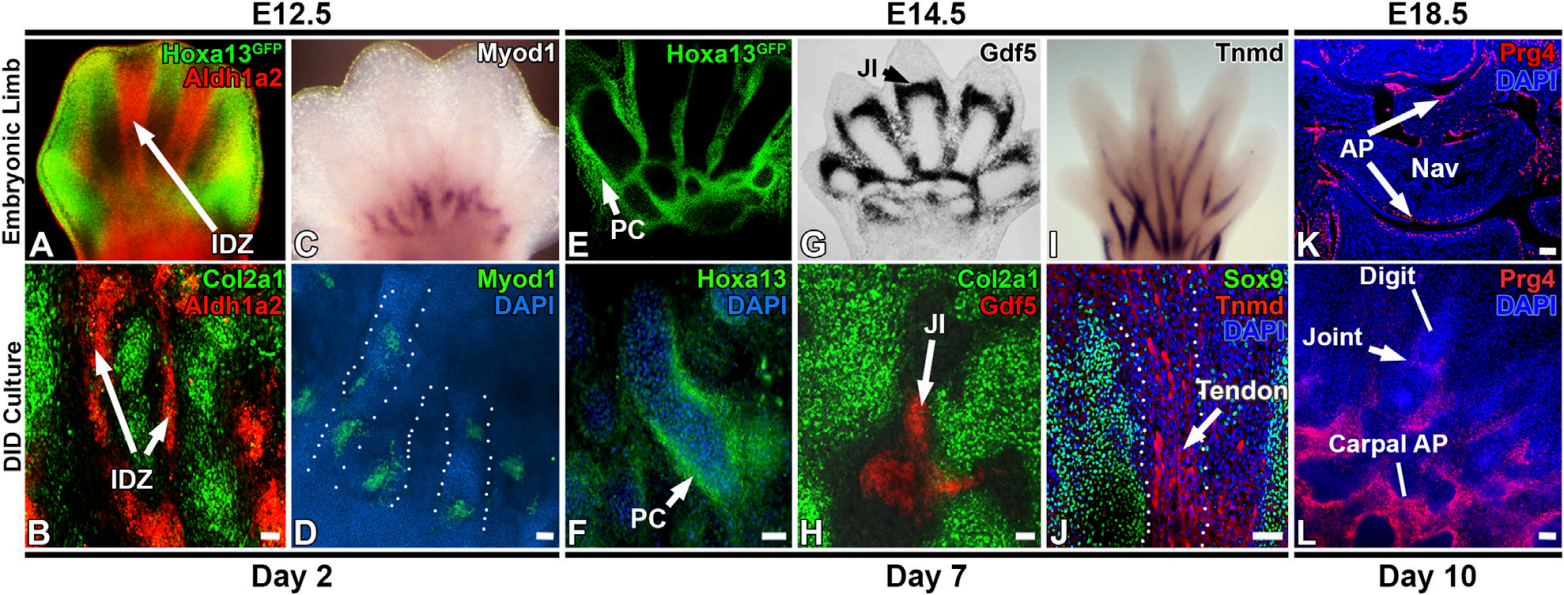
Recapitulation of digit/carpal element gene expression patterns by the DID system. **(A,B)** Day 2 cultures recapitulate digit and interdigital tissue development as detected by *Col2a1* expression in the presumptive digit condensations and *Aldh1a2* in the interdigital zones (arrows, IDZ). **(C,D)** *Myod1* expression pattern at the digit base is recapitulated in Day 2 cultures. Dashed lines depict individual digits forming in the Day DID culture in panel **(D)**. **(E,F)** Day 7 cultures recapitulate *Hoxa13* expression patterns that localize to the periphery of digit condensations. **(G)** Developmental expression of *Gdf5* in the E14.5 forelimb joint interzone (JI, arrowhead). **(H)** Day 7 DID cultures recapitulate *Gdf5* expression localizing to presumptive joint interzones between digit structures (JI, arrow). **(I)** Maturing tendons robustly express *Tnmd* in E14.5 autopods. **(J)** IHC analysis of *Sox9* and *Tnmd* expression in Day 10 DID cultures reveals robust *Tnmd* expression in maturing tendons (arrow) forming between the *Sox9*-expressing digit structures. **(K)** *Prg4* is expressed in the articular perichondrium (AP, arrows) in E18.5 carpal skeletal elements. Nav = navicular element. Rad = radius. **(L)** Day 10 cultures recapitulate the formation of digit, joint, and carpal-like skeletal elements that express *Prg4* in the articular perichondrium (AP) Bars = 50 µm.

The morphologic and transcriptomic similarities between structures forming *in vitro* and in developing digits suggests autopod development is proceeding in the DID system (Figures 2–6, Supplementary Table S2). To test this hypothesis, we examined whether temporally- and spatially-restricted limb developmental gene expression patterns are recapitulated in DID-produced tissues and structures using HCR RNA-FISH and IHC (Choi et al., 2016). Analysis of Day 2 cultures revealed robust expression of *Col2a1* in the digit-like structures similar to the expression pattern seen in E12.5 autopods (Figures 2, 6) (Metsaranta et al., 1995; Bruneau et al., 2001).

Next, the detection of an interdigital cell cluster in the Day 2 scRNAseq analyses (Cluster 11, Figure 3), indicates digit development in the DID cultures includes the formation of interdigital zones, a key signaling tissue that mediates digit identity (Dahn and Fallon, 2000; Huang et al., 2016). To test this hypothesis, we examined the expression of *Aldh1a2*, which is robustly expressed in the interdigital tissues of E12.5 limbs (Figure 6A) (Shou et al., 2013). Analysis of *Aldh1a2* expression in Day 2 cultures revealed a comparable pattern of expression that was restricted to the tissues between the digit-like structures, confirming the development of an interdigital zone by the DID system (Figure 6). Similarly, a myocyte progenitor cluster expressing *Myod1* was also identified in the Day 2 DID cultures (Cluster 12, Figure 3), prompting the hypothesis that autopod muscle development was recapitulated by the DID system. Testing this hypothesis, we characterized the expression of *Myod1* in Day 2 cultures which revealed an expression pattern similar to E12.5 limbs with *Myod1*-expressing cells localized to the base of the digit-like structures (Figure 6) (Hu et al., 2012; Wood et al., 2013).

The detection of *Myod1*-expressing tissues in Day 2 DID cultures indicates that myogenic progenitors are present in the E11.5 limbs bud tissues used to initiate the DID cultures (Figures 2, 6). While *Myod1* expression has not been detected in the distal limb bud at E11.5, the progenitors that ultimately produce *Myod1*-expressing tissue in the limb bud are thought to be present as early as E9.5, as explant cultures using E9.5 limbs robustly produced *Myod1*-expressing tissues after several days of culture (Sassoon et al., 1989). This finding is consistent with our detection of *Myod1*-expressing tissue in Day 2 cultures which we would predict is derived from pre-existing myogenic progenitors in the dissected E11.5 limb tissues used to establish the DID cultures (Figures 3, 6, and Supplementary Table S2).

In Day 7 cultures, scRNAseq analysis identified enrichment of *Jun*, *Gdf5*, and *Hoxd13* expression in Cluster 4, suggesting the formation of a joint interzone in these cultures (Figure 4) (Storm and Kingsley, 1999; Kan and Tabin, 2013; Huang et al., 2016; Capellini et al., 2017). Testing this hypothesis, we examined the expression of *Gdf5* in Day 7 DID cultures which revealed robust expression between digit segments in a pattern similar to joint interzone regions present in E14.5 limbs, confirming the formation of joint interzones in the Day 7 DID cultures (Figure 6) (Storm and Kingsley, 1999; Capellini et al., 2017). Day 7 DID cultures also recapitulated the *Hoxa13* expression pattern seen in E14.5 limbs where it localizes to the periphery of the digit-like structures (Figure 6) (Stadler et al., 2001; Villavicencio-Lorini et al., 2010; Shou et al., 2013). Characterization of *Tnmd* expression in Day 7 DID cultures revealed elongated tendon-like structures robustly expressing this mature tendon marker, recapitulating the expression seen in E14.5 autopods (Figure 6) (Shukunami et al., 2018).

Evaluation of *Col1a1* and *Thbs2* expression in Day 10 DID cultures revealed restricted expression to the flattened cell sheets surrounding the digit- and carpal-like structures, confirming the formation of a perichondrium (Figure 2). Finally, we assessed whether the digit-like tissues present in the Day 10 DID system also produce an articular perichondrium, a specialized tissue found at the epiphyses of late embryonic and postnatal skeletal elements that contributes to cells forming the articular cartilage and portions of the synovial joint capsule (Kozhemyakina et al., 2015; Zhang et al., 2021). A canonical marker of articular perichondrium, *Prg4*, was identified as robustly expressed by cells assigned to Cluster 3 in the Day 10 datasets, suggesting this tissue may be present in the Day 10 cultures (Figure 5; Supplementary Figure S4). Analysis of *Prg4* expression in Day 10 cultures and E18.5 limbs confirmed localization of this marker to the articular perichondrium surrounding the developing carpal elements, and at the base of the developing digits, with faint expression also detected in a presumptive joint field (Figure 6). This expression pattern indicates that articular cartilage development is proceeding in the Day 10 DID cultures.

### 3.9 Recapitulation of *Hoxa13* mutant limb defects in the DID system

**FIGURE 7:**
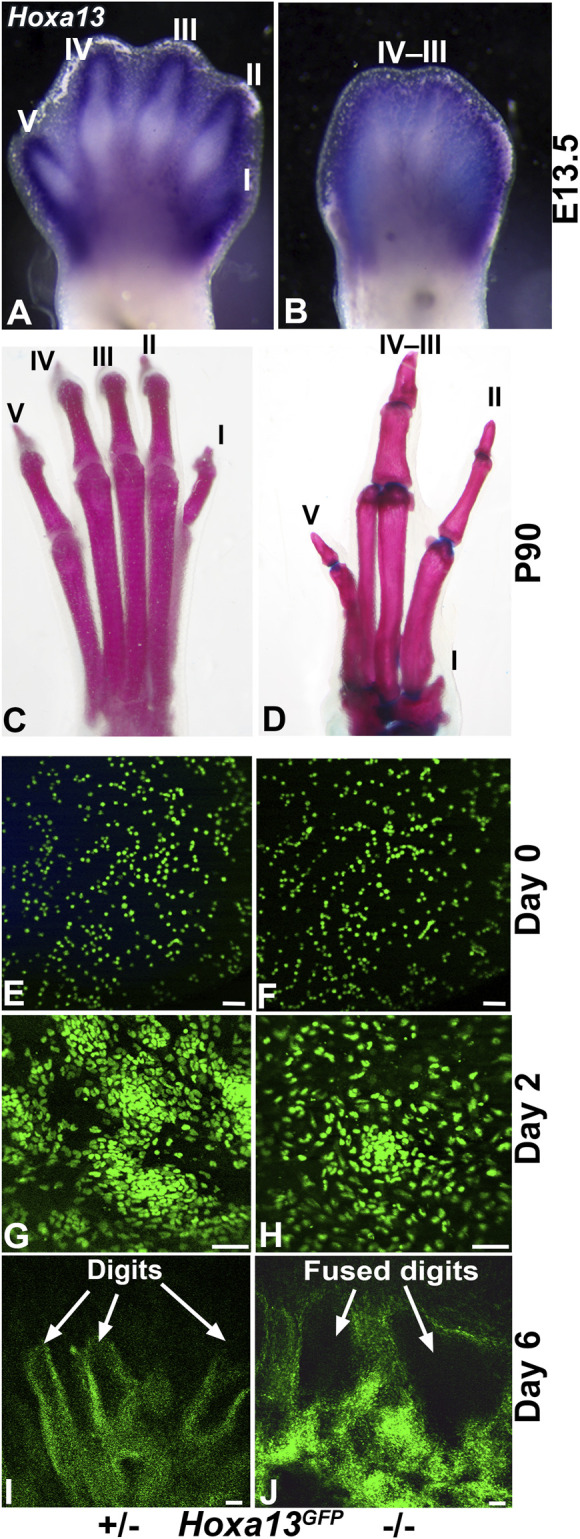
Recapitulation of the *Hoxa13* mutant limb defects by the DID system. **(A)** *Hoxa13* is robustly expressed in forelimb and hindlimb digits at E13.5 in *Hoxa13* heterozygous mutant embryos. **(B)** *Hoxa13* *^GFP^* homozygous mutant embryos exhibit poor separation of the hindlimb digits by E13.5. **(C, D)** Comparison of postnatal day 90 (P90) littermate hindlimbs reveals normal development of digits in heterozygous *Hoxa13* *^GFP^* mice compared to homozygous mutants that exhibit loss of digit I, fusion of digit III-IV phalangeal elements, and brachydactyly and phalangeal element loss in digit V. Postnatal *Hoxa13* *^GFP^* limb defects. **(E, G, I)** DID culture of *Hoxa13* *^GFP^* heterozygous mutant distal limb mesenchyme reveals normal mesenchymal condensation at Day 2 (panel **G**) and production of multiple digit-like structures by Day 6. **(F, H, J)** DID culture of *Hoxa13* *^GFP^* homozygous mutant mesenchyme reveals delayed mesenchymal condensation at Day 2 (panel **H**) followed by the production of several fused digits (panel **J**). Bars = 50 µm for panels **E–J**.

Mutations in *Hoxa13* profoundly affect distal limb development causing reduced mesenchymal condensation, loss of digit I, as well as fusion of hindlimb digits III and IV (Figure 7) (Fromental-Ramain et al., 1996; Stadler et al., 2001; Perez et al., 2010). Recognizing the capacity of the DID system to recapitulate distal limb development, we hypothesized that it could be used to model the *Hoxa13* mutant limb phenotypes (Figure 7) (Fromental-Ramain et al., 1996; Stadler et al., 2001; Perez et al., 2010). To test this hypothesis, distal hindlimb mesenchyme expressing *Hoxa13* was isolated from heterozygous and homozygous mutant embryos using fluorescence activated cell sorting (FACS) and the *Hoxa13* ^*GFP*^ allele as described (Stadler et al., 2001). DID culture of FACS-enriched *Hoxa13* ^*GFP*^ distal limb mesenchyme from heterozygous E11.5 embryos produced numerous cell condensations by Day 2 (n = 3/3) (Figure 7). By Day 6, heterozygous DID cultures exhibited robust formation of digit-like structures similar to those produced by wild type distal limb mesenchyme (n = 3/3) (Figures 2, 7). In contrast, homozygous mutant cells placed in DID culture exhibited a delay in condensation, producing loosely organized structures in Day 2 cultures (n = 4/4) (Figure 7). By Day 6, homozygous mutant DID cultures produced noticeably larger digit-like condensations resembling digit fusions seen in homozygous mutant hindlimbs (Figure 7) (n = 3/4). Taken together these results indicate that defects in mesenchymal condensation and digit fusions exhibited by *Hoxa13* mutant mice are recapitulated by the DID system.

## 4 Discussion

Analysis of DID cultures indicates robust development of distal limb tissues and structures including digits, joints, perichondrium, muscles, and tendons. An essential question for the applicability of the DID system to study distal limb development is whether it effectively models stage-specific molecular and cellular processes mediating digit and joint formation. Recognizing that undifferentiated distal limb mesenchyme from E11.5 embryos was used to inoculate the DID cultures (Day 0, Figure 2), we predicted that progressive autopod development would be evident if DID cultures exhibit patterns of gene expression similar to limbs older than E11.5.

Progressive autopod development was initially confirmed in Day 2 cultures which reproduced gene expression patterns for *Aldh1a2* and *Myod1* similar to E12.5 autopods (Figure 6) (Anderson et al., 2012; Shou et al., 2013). Assignment of an E12.5 equivalency to the Day 2 time point is also supported by recent scRNAseq studies of E12.5 limbs that identified the same cell lineages as detected in our Day 2 scRNAseq datasets which included cell types annotated as muscle lineages expressing *Myod1* or as distal mesenchyme expressing *Aldh1a2* and *Hoxd13* (Figures 3, 6; Supplementary Figure S2, and Supplementary Table S2) (Desanlis et al., 2020).

Progressive development of the autopod tissues beyond E12.5 was also evident in the Day 7 DID cultures which exhibited tissues and gene expression patterns similar to E13.5–14.5 autopods including: localization of *Hoxa13-*expressing cells to developing digit perichondrium, formation of joint interzones that express *Gdf5*, and the formation of tendon-like structures expressing *Tnmd* (Figure 6) (Storm and Kingsley, 1999; Stadler et al., 2001; Shou et al., 2013; Shwartz et al., 2016; Shukunami et al., 2018). Gene expression patterns present in Day 10 cultures were also indicative of additional developmental progression of the autopod tissues which we estimated to be E18.5-Postnatal Day 0, based on the expression pattern of *Prg4* in the carpal and digit articular perichondrium which is generally first detected early postnatal limbs (Kozhemyakina et al., 2015; Zhang et al., 2021).

Taken together, we conclude the DID system provides a progressive model of murine autopod development, recapitulating stage-specific formation of musculoskeletal tissues and structures. An important caveat to the DID system is that the progressive development of autopod tissues appears delayed by one (Day 2 cultures) to three days (Day 10 cultures) compared to the chronological age of the cells used to inoculate DID system. We attribute this heterochrony to the time required for dissociated E11.5 mesenchyme to reassemble and reinitiate an autopod developmental program *in vitro*.

### 4.1 New insights into models of proximal-distal limb development

In vertebrates, limb development proceeds sequentially, producing skeletal elements in a proximal to distal (P-D) manner, forming the humerus first, followed by ulna/radius, carpals, metacarpals, and finally phalangeal elements (Saunders, 1948; Summerbell et al., 1973; Tickle et al., 1975; Wolpert et al., 1975; Wolpert, 1990). While it is well-established that the apical ectodermal ridge (AER) and its production of FGFs regulate P-D limb bud outgrowth, additional studies are required to define the cellular and molecular mechanisms mediating P-D patterning and sequential development of limb skeletal elements (Saunders, 1948; Niswander et al., 1993; Cohn et al., 1995; Lewandoski et al., 2000; Dudley et al., 2002; Sun et al., 2002). To date, two models have emerged to explain how positional cell identity is established in the undifferentiated mesenchyme to facilitate P-D patterning of limb skeletal elements.

In the progress zone model, P-D specification of limb structures is determined by the time cells spend in the progress zone (PZ), an undifferentiated region of mesenchyme located in the distal limb bud (Summerbell et al., 1973; Summerbell and Wolpert, 1973). Cells spending the shortest amount of time in the PZ are specified to form proximal limb structures, whereas cells spending longer periods in the PZ form distal structures, with AER-derived FGFs functioning as the key mediator of cell specification (Summerbell and Wolpert, 1973; Tickle and Wolpert, 2002). By this model, the recapitulation of carpal and digit structures by DID cultures using PZ mesenchyme would indicate that distal structure specification is active in the DID system, requiring, at a minimum, continued expression of *Fgf4* or *Fgf8*. Analysis of *Fgf4* and *Fgf8* expression in the Day 2 cultures revealed no cells actively expressing these genes (Supplementary Table S2). This result indicates that the recapitulation of distal limb development by the DID cultures may not follow the progress zone model.

One explanation for DID cultures producing distal structures in the absence of *Fgf4* and *Fgf8* expression is the presence of FGFs in fetal calf serum used in the DID culture media. In this scenario, FGF-mediated specification of distal limb structures would be facilitated if cells used in the Day 2 DID cultures express FGF receptors. This possibility is supported by the Day 2 scRNAseq analysis that identified expression of *Fgfr1*, *Fgfr2*, and *Fgfr3* in cells annotated as chondrocytes in Clusters 1, 7, 9, and 13 (Figure 3 and Supplementary Table S2). However, while the bovine genome contains *Fgf4* and *Fgf8* orthologs, these proteins are not generally included as the endocrine FGFs present in fetal calf serum, reducing the possibility that serum-supplied FGF4 or FGF8 mediates distal limb specification to support a progress zone model of distal limb development in DID cultures (Itoh, 2010; Itoh et al., 2015; Itoh et al., 2016).

A second model suggests that P-D patterning is specified early in the distal limb mesenchyme, allowing structures to differentiate sequentially as the limb grows out under the influence of the AER (Dudley et al., 2002; Sun et al., 2002). A critical feature of the early specification model is that lineage-restricted cell compartments should be present in the distal mesenchyme. By this model, the preferential recapitulation of carpal and digit development by DID cultures should be reflected by the presence of distally-restricted cell populations.

Lineage tracing in the developing limbs has confirmed the presence of restricted cellular compartments establishing the dorsal-ventral axis (Arques et al., 2007). However, similar analyses of the P-D axis revealed a mixture of *Hoxa11*- and *Hoxa13*-expressing cells in the distal limb (Sato et al., 2007). Based on the detection of the proximal zeugopod marker, *Hoxa11*, in a cell mixture with *Hoxa13* in the distal autopod prompted the conclusion that the early specification model may not be a well-suited mechanism for autopod patterning.

This conclusion may be premature as more recent studies have identified distally-restricted *Hoxa11* antisense long non-coding RNAs functioning as primary mediators of tetrapod digit development (Kherdjemil et al., 2016; Leite-Castro et al., 2016). Analysis of the Day 2 scRNAseq datasets identified *Hoxa11-* and *Hoxa13-*expressing cells in Cluster 6 along with expression of the *Hoxa11* antisense gene, *Hoxa11os*, supporting the premise that early specification of distal limb structures includes expression of *Hoxa11* and its antisense long non-coding RNAs (Supplementary Table S2).

For DID cultures to further elucidate whether progress zone- or early specification models mediate P-D patterning of limb mesenchyme, we anticipate several future experiments. Notably, it will be essential to assess P-D patterning in DID cultures using defined growth media. By this approach, we can discern whether distal patterning of DID cultures reflects active specification of progress zone cells by exogenous FGF signaling. Next, to discern the mechanisms mediating P-D patterning in the early specification model, it will be essential to identify the role of cell surface proteins in the establishment of cell positional identity. In this context, DID cultures could be used to interrogate individual and combinatorial cell surface protein functions mediating sorting and compartmentalization of proximally- and distally-fated cell lineages.

### 4.2 DID cultures as autopod organoids

A major question regarding the DID cultures is whether they represent an autopod organoid? Traditionally, organoids were defined as tissues or structures that develop *in vitro* from stem cells or tissue progenitors using cell sorting and spatially-restricted lineage commitment to self-organize into an organ-like structure (Lancaster and Knoblich, 2014). By this definition, the DID cultures could be considered an autopod organoid as the cells dissociated from the distal mesenchyme self-assemble and organize into spatially-restricted lineages that differentiate into discrete musculoskeletal structures including digits, carpal elements, joints, muscles, perichondrium, and tendon. This designation is supported by the scRNAseq analysis of the DID cultures which detected tenocyte, myocyte, stromal, and chondrocyte progenitor populations (Figures 3–5; Supplementary Table S2).

More recently, organoids have been redefined as three-dimensional structures grown *in vitro* from stem cells that self-organize through cell sorting and spatially-restricted lineage commitment to produce organ-specific cell types (Clevers, 2016). Using these revised criteria, the DID system should not be considered an organoid, as a stem cell population was not used or identified as the facilitating cell type mediating robust recapitulation of hand/foot development in the DID cultures.

### 4.3 Do immune cell populations in the DID cultures contain osteoclasts?

Cells annotated in the DID cultures as immune cells (Figures 3–5) may also include osteoclasts, a specialized musculoskeletal cell type often thought of as the immune component of the endochondral bone (Sato et al., 2006; Schaffler et al., 2014). The progressive development of digit and carpal elements in the DID cultures support this possibility, as osteoclasts function to remodel bone to facilitate developmental growth (Sato et al., 2006; Schaffler et al., 2014; Yahara et al., 2020; Yahara et al., 2022). Interestingly, the same canonical factors used to annotate the immune cell lineages in the DID scRNAseq datasets (Figures 3–5) are also used to distinguish osteoclasts including: *Tyrobp*, *Trem2*, and *C1qa*, *C1qb*, and *C1qc*, supporting the possibility that a portion of the immune cell types present in the DID cultures may be osteoclasts (Figures 3–5) (Sato et al., 2006; Teo et al., 2012; Xia et al., 2017; Chen et al., 2021; Liang et al., 2021; Dong et al., 2022). This conclusion is supported by the developmental origins of some osteoclast lineages, which are derived from yolk sac erythromyeloid progenitors present in the embryo prior to E10.5 (Lux et al., 2008; Yahara et al., 2020; Yahara et al., 2022).

### 4.4 DID cultures partially model limb vasculogenesis

A key step to producing a mature vascular network in the vertebrate limb is the initial formation of a rudimentary vascular plexus derived from cells emerging from the dorsal aorta (Seichert and Rychter, 1971; 1972). The absence of an aortic source for these cells in the DID cultures provides a likely reason for the lack of *Pecam1* (*Cd31*) expression, as endothelial maturation requires the formation of a vascular plexus which is remodeled into a mature limb vascular network (Eshkar-Oren et al., 2009; Takimoto et al., 2009). It is interesting to speculate whether co-culture of dissociated limb bud mesenchyme with aortic tissues in DID cultures would facilitate the formation of the initial vascular plexus and a mature vascular network.

In maturing endochondral bones, vascular invasion is facilitated by the expression of *Vegfa* by hypertrophic chondrocytes which attracts endothelial and osteclast cell types from blood vessels proximal to the bone sheath (Takimoto et al., 2009; Kusumbe and Adams, 2014). Our detection of the hypertrophic chondrocyte marker, *Col10a1*, in the same clusters as *Vegfa* in Day 10 cultures (Clusters 6 and 9) suggests that the initial step to attract endothelial and osteoclastic cell types to the digit tissues occurs independent of vascular involvement and is modeled by the DID system (Supplementary Figures S6, S7, and Supplementary Table S2). As a vascular source is not present in the DID cultures, it is likely that endothelial cells detected by the scRNAseq analysis are derived from multiple sources including: *Cd34+* mesenchymal stem cells, endothelial cells resident to the perichondrium, or from endothelial progenitors that migrate into the limb bud as early as E9.5 (Colnot et al., 2004; Tozer et al., 2007; Yvernogeau et al., 2012; Carbone et al., 2016; AbuSamra et al., 2017).

### 4.5 Modeling opportunities

A prominent feature of the DID system is its capacity to model congenital defects caused by the loss of *Hoxa13* function (Figure 6). While many of the transcriptional targets of *Hoxa13* have been identified in E11–12.5 limbs, the cellular mechanisms regulated by *Hoxa13* to facilitate digit patterning remain poorly understood (Knosp et al., 2004; Knosp et al., 2007; Shou et al., 2013; Kherdjemil et al., 2016; Sheth et al., 2016; Bastida et al., 2020; Desanlis et al., 2020; Trofka et al., 2021). In this context, the modeling of congenital defects by the DID system provides a novel tool to identify cell-specific functions of target genes that regulate the formation and pattering of digit tissues.

As *Hoxa13* was recently identified as a primary mediator of digit regeneration in urodeles (Takeuchi et al., 2022), it is interesting to speculate whether distal mesenchyme from regenerative species could be used in the DID system to rapidly identify and validate *Hoxa13* regenerative target genes. Once identified, these regenerative factors could also be evaluated as therapeutics to improve digit regeneration in mammals, whose regenerative capacity is currently limited to the distal digit tip (Han et al., 2008; Dawson et al., 2018; Dolan et al., 2018; Yu et al., 2019; Johnson et al., 2020). Recent studies investigating the regenerative capacity of more proximal digit amputations in mice indicate that the remaining tissues are competent to regenerate bone and joint tissues when provided with sequential applications of BMP2 and BMP9 (Yu et al., 2019). In this context, *in vitro* digit amputations created in the DID system could be used to rapidly develop sequential and/or combinatorial treatments to stimulate the complete regeneration of digit tissues.

The formation of a perichondrium by DID skeletal elements provides an additional opportunity to examine how periosteal tissues participate in the repair and homeostasis of bone. Recent lineage tracing studies have identified a *Dlx5*-expressing cell population in the outer fetal perichondrium as a primary cell-of-origin for postnatal bone marrow stroma with an adipocyte biased state (Matsushita et al., 2022). Upon injury, adipocyte biased cells rapidly convert to a skeletal cell state to facilitate bone repair (Matsushita et al., 2022). These findings suggest that the DID system could be used to rapidly identify factors that increase *Dlx5*-expressing cell populations in the outer perichondrium, providing an approach to develop new treatments for non-union fractures.

In summary, our analysis of the DID cultures indicates robust recapitulation of murine autopod development. Access provided by the DID system to discrete cell populations as they form specialized tissues and structures of the hand and foot represents an important advancement in how studies can be designed to interrogate the cellular and molecular mechanisms mediating limb development. As the DID system can also reproduce congenital defects, it is anticipated that this system will facilitate the development of new therapies aimed at discerning the molecular pathology of specific malformations as well as providing a new tool to identify effective therapies to stimulate the repair/regeneration of hand/foot musculoskeletal tissues impacted by congenital malformation, injury or disease.

## Data Availability

The datasets presented in this study can be found in online repositories. The names of the repository/repositories and accession number(s) can be found below: Gene Expression Omnibus accession number GSE221883.
