# Supplementary material for: Digits in a dish: An in vitro system to assess the molecular genetics of hand/foot development at single-cell resolution
Source: Front Cell Dev Biol. 2023 Mar 13;11:1135025. doi: 10.3389/fcell.2023.1135025 (PMC10040768; doi:10.3389/fcell.2023.1135025)
Supplement: Supplementary file 1 [file DataSheet1.zip › Supplementary Data/Supplementary Materials Legends.docx]

**10 Supplemental Materials**

**Figure S1.** **Distribution of cells from replicates across cell cluster populations.** (**A**) UMAP (Uniform Manifold Approximation and Projection) plot of Day 2 culture scRNAseq results denoting Replicate A (pink) and Replicate B (blue). (**B**) UMAP plots of Day 2 culture split by Replicate A (left) and Replicate B (right). Individual cells are color coded based on cluster. (**C**) UMAP (Uniform Manifold Approximation and Projection) plot of Day 2 culture scRNAseq results denoting Replicate A (pink) and Replicate B (blue). (**D**) UMAP plots of Day 2 culture split by Replicate A (left) and Replicate B (right). Individual cells are color coded based on cluster. (**E**) UMAP (Uniform Manifold Approximation and Projection) plot of Day 2 culture scRNAseq results denoting Replicate A (pink) and Replicate B (blue). (**F**) UMAP plots of Day 2 culture split by Replicate A (left) and Replicate B (right). Individual cells are color coded based on cluster. Percentage of cells contributing to the composition of the individual clusters from Replicates A and Replicates B are listed to the right of cluster legend.

**Figure S2.** **Day 2 pattern of marker expression split by replicates.** Split dot plots highlighting expression profiles of selected marker genes per cell type used for cluster cell identification split by replicate. Red dots cells are from Day 2, Replicate A, Blue dots are from Day 2, Replicate B. Dot diameter corresponds to the percentage of cells expressing the gene in each cluster. High-Low expression is denoted by Blue-Gray- and Red-Gray bars for replicates A and B respectively.

**Figure S3.** **Day 7 pattern of marker expression split by replicates.** Split dot plots highlighting expression profiles of selected marker genes per cell type used for cluster cell identification split by replicate. Red dots cells are from Day 7, Replicate A, Blue dots are from Day 7, Replicate B. Dot diameter corresponds to the percentage of cells expressing the gene in each cluster. High-Low expression is denoted by Blue-Gray- and Red-Gray bars for replicates A and B respectively.

**Figure S4.** **Day 10 pattern of marker expression split by replicates.** Split dot plots highlighting expression profiles of selected marker genes per cell type used for cluster cell identification split by replicate. Red dots cells are from Day 10, Replicate A, Blue dots are from Day 10, Replicate B, Dot diameter corresponds to the percentage of cells expressing the gene in each cluster. High-Low expression is denoted by Blue-Gray- and Red-Gray bars for replicates A and B respectively.

**Figure S5. scRNAseq analysis of *Epha7* expression in DID cultures.**

Violin plot representations of Cluster-specific cell expression of *Epha7* in Day 2 DID cultures (**A**), Day 7 DID cultures (**B**), and Day 10 DID cultures (**C**). Asterisks denote significant differential gene expression as listed in Supplemental Table 2. Significant differential expression was determined as described in the Methods Section using the FindAllMarkers function that applied a Wilcoxon rank sum test with a minimum 0.25 log fold change between clusters and expressed in at least 25% of cells from the cluster.

**Figure S6. scRNAseq analysis of endothelial marker expression in DID cultures.**

(**A**) Violin plot representation of Cluster-specific cell expression of *Cd34* expression in Day 2, 7, and 10 DID cultures. (**B**) Violin plot representation of Cluster-specific cell expression of *Icam1* expression in Day 2, 7, and 10 DID cultures. (**C**) Violin plot representation of Cluster-specific cell expression of *Kdr* expression in Day 2, 7, and 10 DID cultures. (**D**) Violin plot representation of Cluster-specific cell expression of *Pecam1* (*Cd31*) expression in Day 2, 7, and 10 DID cultures. (**E**) Violin plot representation of Cluster-specific cell expression of *Tek* (*Tie2*) expression in Day 2, 7, and 10 DID cultures. (**F**) Violin plot representation of Cluster-specific cell expression of *Vcam1* expression in Day 2, 7, and 10 DID cultures. Asterisks denote significant differential gene expression as listed in Supplemental Table 2. Significant differential expression was determined as described in the Methods Section using the FindAllMarkers function that applied a Wilcoxon rank sum test with a minimum 0.25 log fold change between clusters and expressed in at least 25% of cells from the cluster.

**Figure S7. scRNAseq analysis of Vasculogenic markers in DID cultures.** (**A**) Violin plot representation of Cluster-specific expression of *Col10a1* in Day 2, 7, and 10 DID cultures. (**B**) Violin plot representation of Cluster-specific expression of *Vegfa* expression in Day 2, 7, and 10 DID cultures. (**C**) Violin plot representation of Cluster-specific expression of *Flt1* expression in Day 2, 7, and 10 DID cultures. Asterisks denote significant differential gene expression as listed in Supplemental Table 2. Significant differential expression was determined as described in the Methods Section using the FindAllMarkers function that applied a Wilcoxon rank sum test with a minimum 0.25 log fold change between clusters and expressed in at least 25% of cells from the cluster.

**Figure S8. scRNAseq analysis of Dorsal-Ventral limb developmental markers in DID cultures.** (**A**) Violin plot representation of Cluster-specific cell expression of *Wnt7a* expression in Day 2, 7, and 10 DID cultures. N.D.= not detected. (**B**) Violin plot representation of Cluster-specific cell expression of *En1* expression in Day 2, 7, and 10 DID cultures. (**C**) Violin plot representation of Cluster-specific cell expression of *Lmx1b* expression in Day 2, 7, and 10 DID cultures. Asterisks denote significant differential gene expression as listed in Supplemental Table 2. Significant differential expression was determined as described in the Methods Section using the FindAllMarkers function that applied a Wilcoxon rank sum test with a minimum 0.25 log fold change between clusters and expressed in at least 25% of cells from the cluster.

**Figure S9. scRNAseq analysis of Anterior limb developmental marker *Asb4* in DID cultures.** Violin plot representation of Cluster-specific cell expression of *Asb4* expression in Day 2 DID cultures (**A**), Day 7 DID cultures (**B**), and Day 10 cultures (**C**).

**Supplementary Table S1.** **Sequencing statistics and parameters used for processing.**

**Supplementary Table S2.** **Differentially Expressed Markers for Cell Clusters by Time Point.** List of the positive, differentially expressed genes using the FindAllMarkers function in Seurat for the Day 2 integrated analysis (**Tab A**), Day 7 integrated analysis (**Tab B**), Day 10 integrated analysis (**Tab C**). Column headers include Gene (NCBI gene ID), p val (unadjusted p-value), avg log_2_FC (average log_2_ fold-change among cell clusters), pct1 (percentage of cells in the cluster expressing the corresponding gene), pct2 (percentage of cells in all clusters expressing the corresponding gene). p val adj (Bonferroni corrected p-value), and cluster (cell cluster number on the associated UMAP plots).
