# Supplementary figures and images for: Digits in a dish: An in vitro system to assess the molecular genetics of hand/foot development at single-cell resolution
Source: Front Cell Dev Biol. 2023 Mar 13;11:1135025. doi: 10.3389/fcell.2023.1135025 (PMC10040768; doi:10.3389/fcell.2023.1135025)

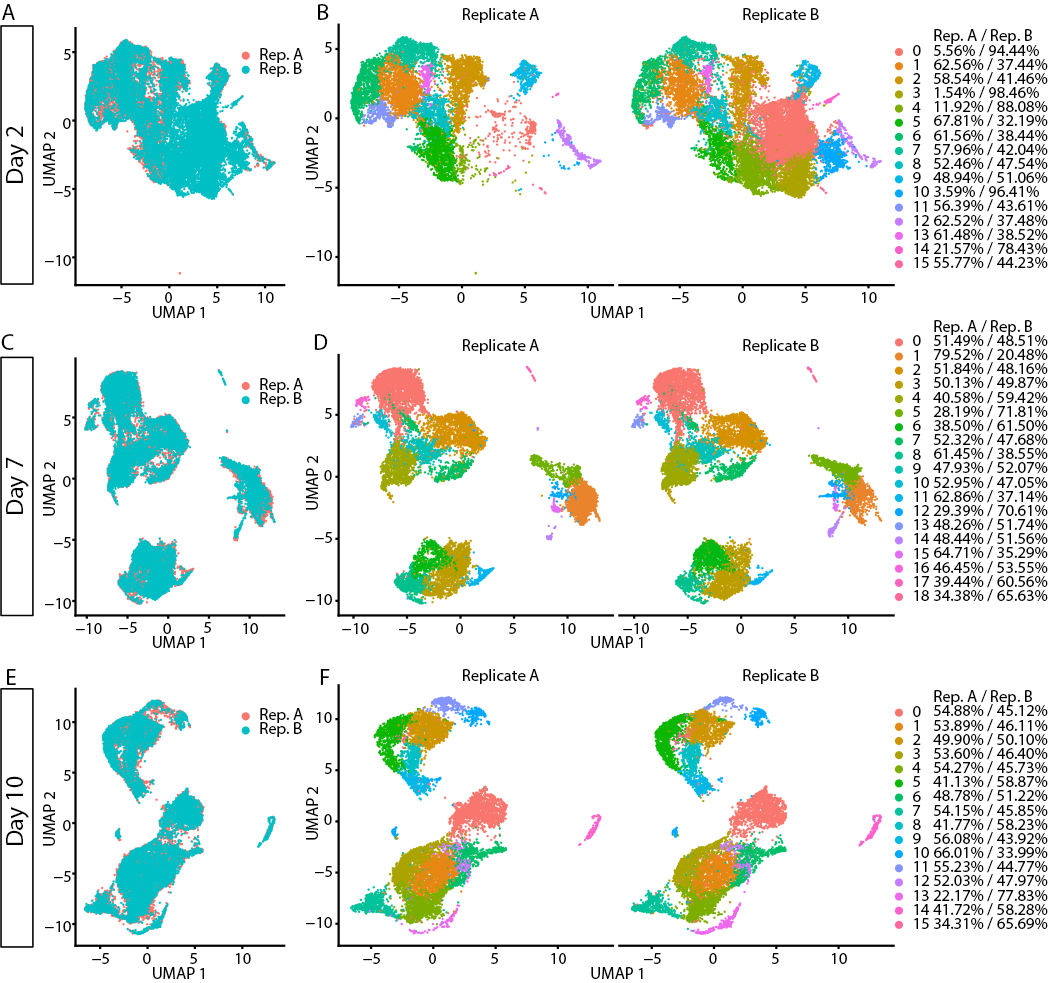

Supplement: Supplementary file 1 [file DataSheet1.zip › Supplementary Data/Supplementary Figure 1.TIF]

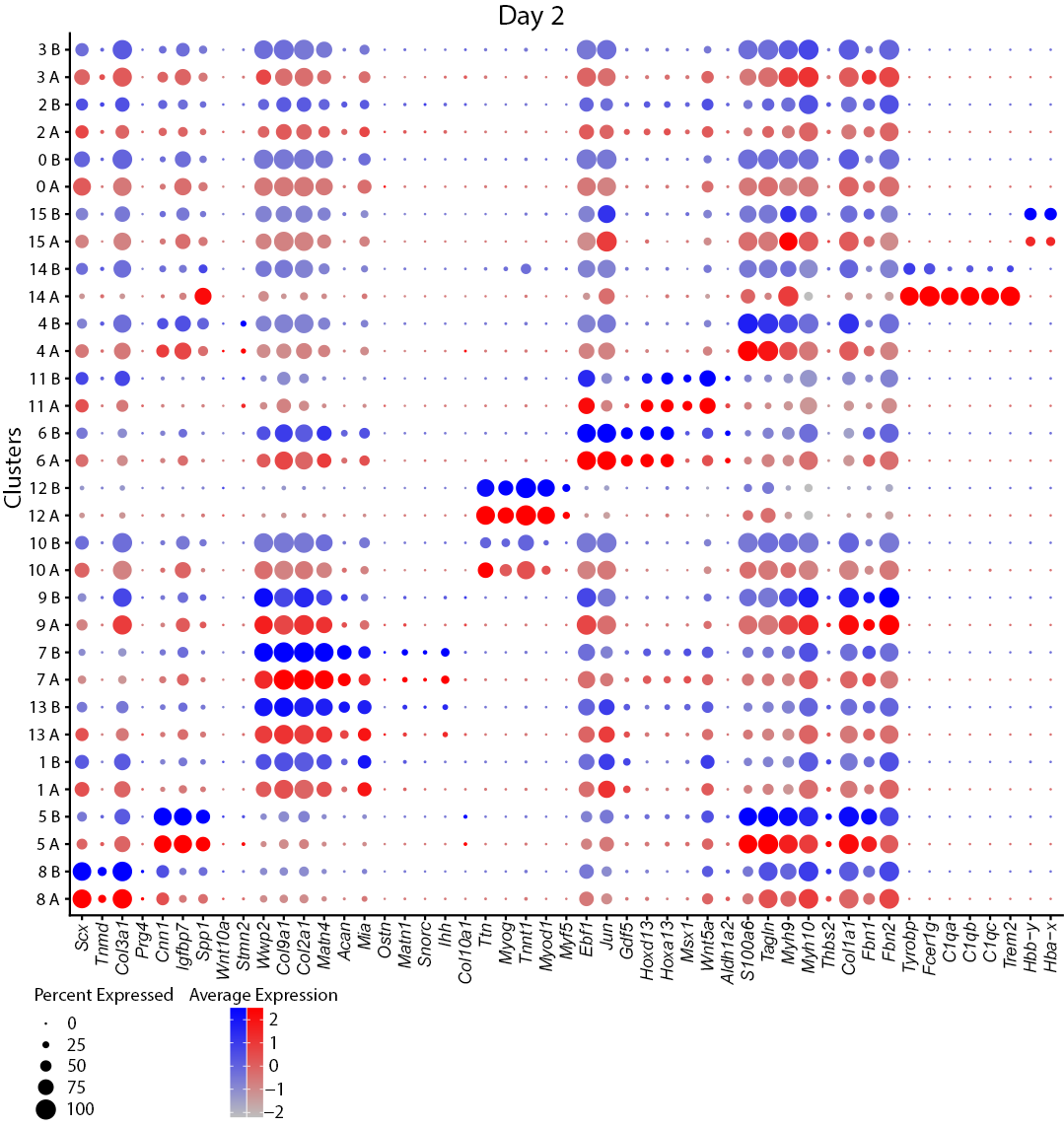

Supplement: Supplementary file 1 [file DataSheet1.zip › Supplementary Data/Supplementary Figure 2.TIF]

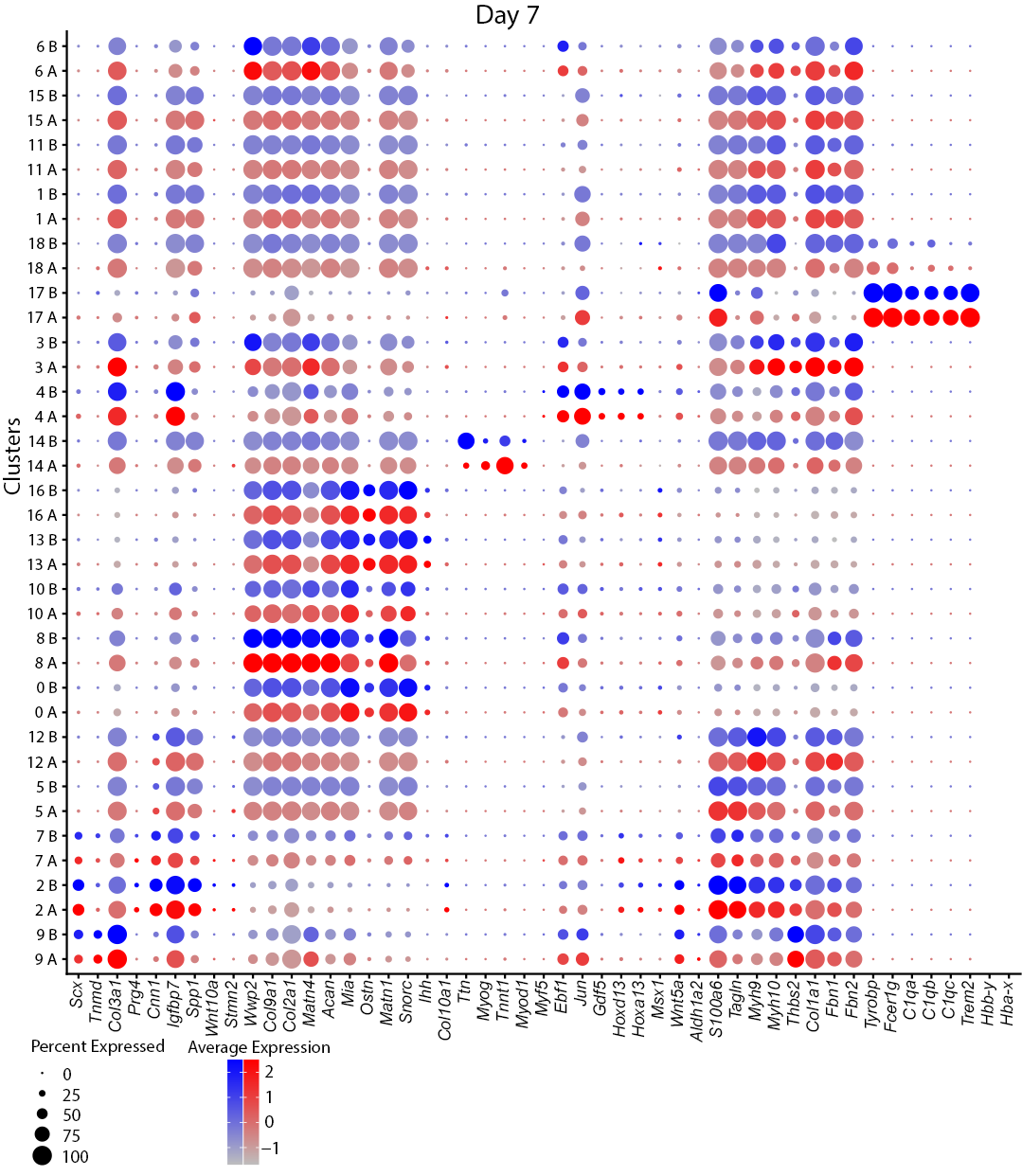

Supplement: Supplementary file 1 [file DataSheet1.zip › Supplementary Data/Supplementary Figure 3.TIF]

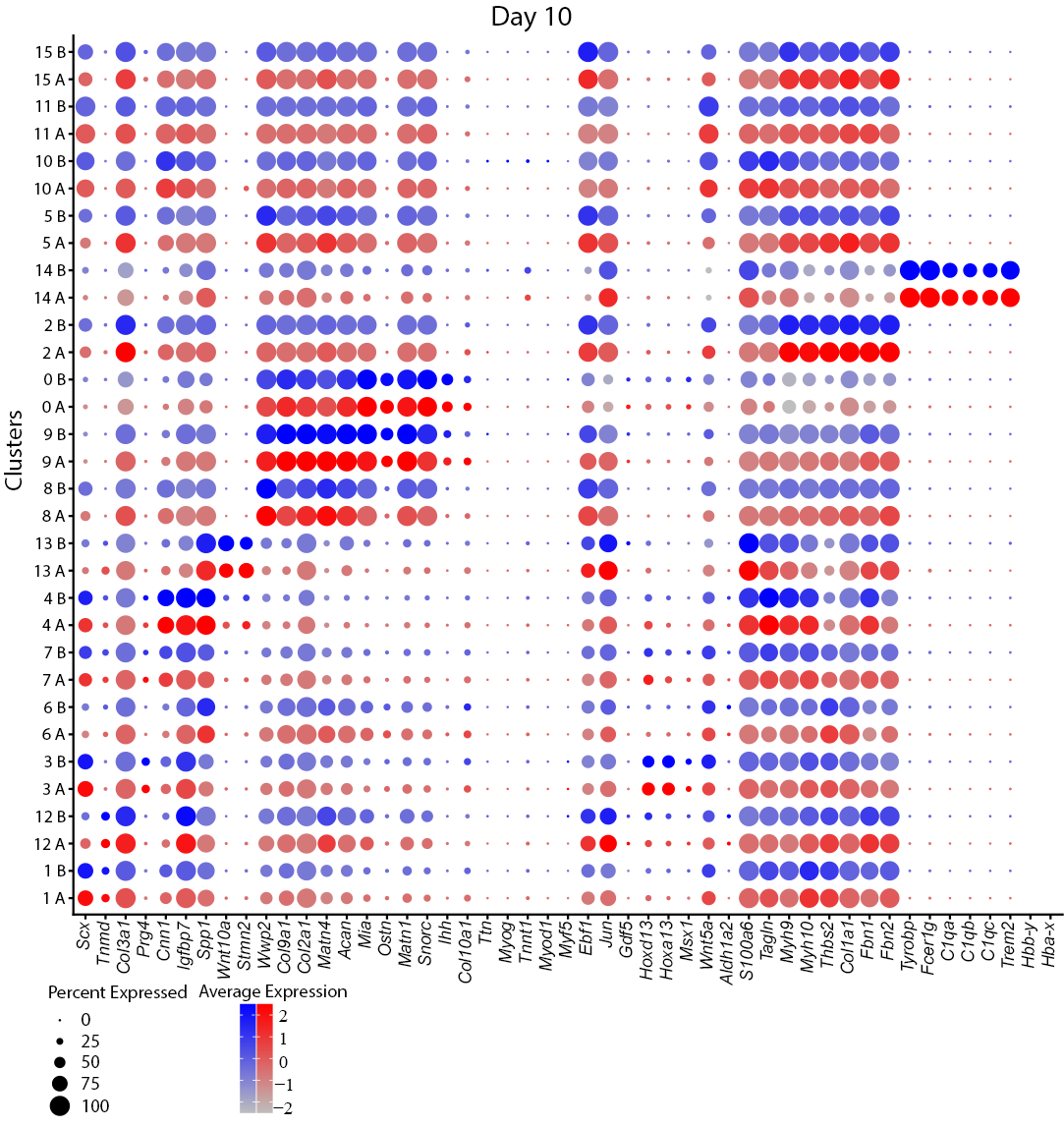

Supplement: Supplementary file 1 [file DataSheet1.zip › Supplementary Data/Supplementary Figure 4.TIF]

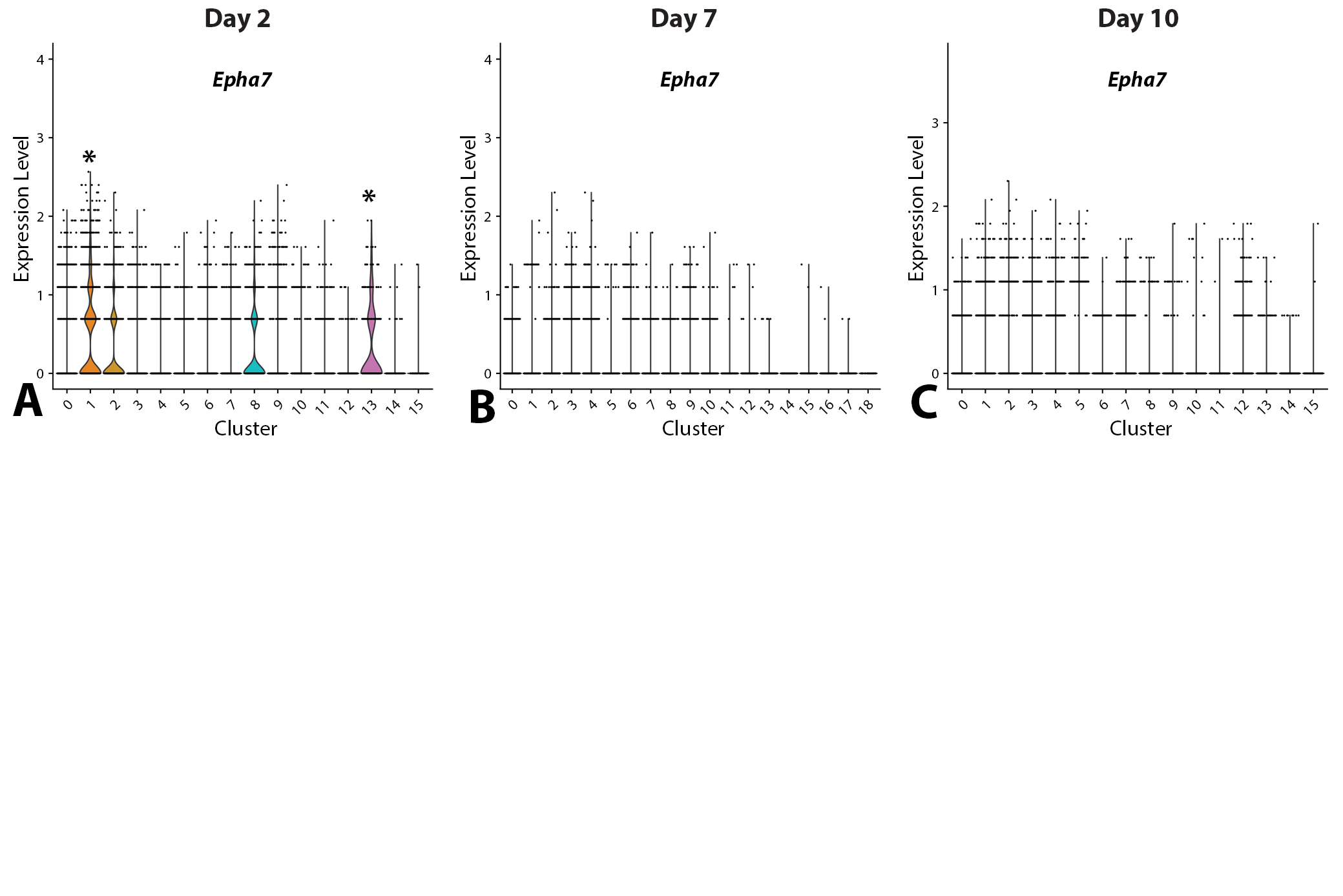

Supplement: Supplementary file 1 [file DataSheet1.zip › Supplementary Data/Supplementary Figure 5.TIF]

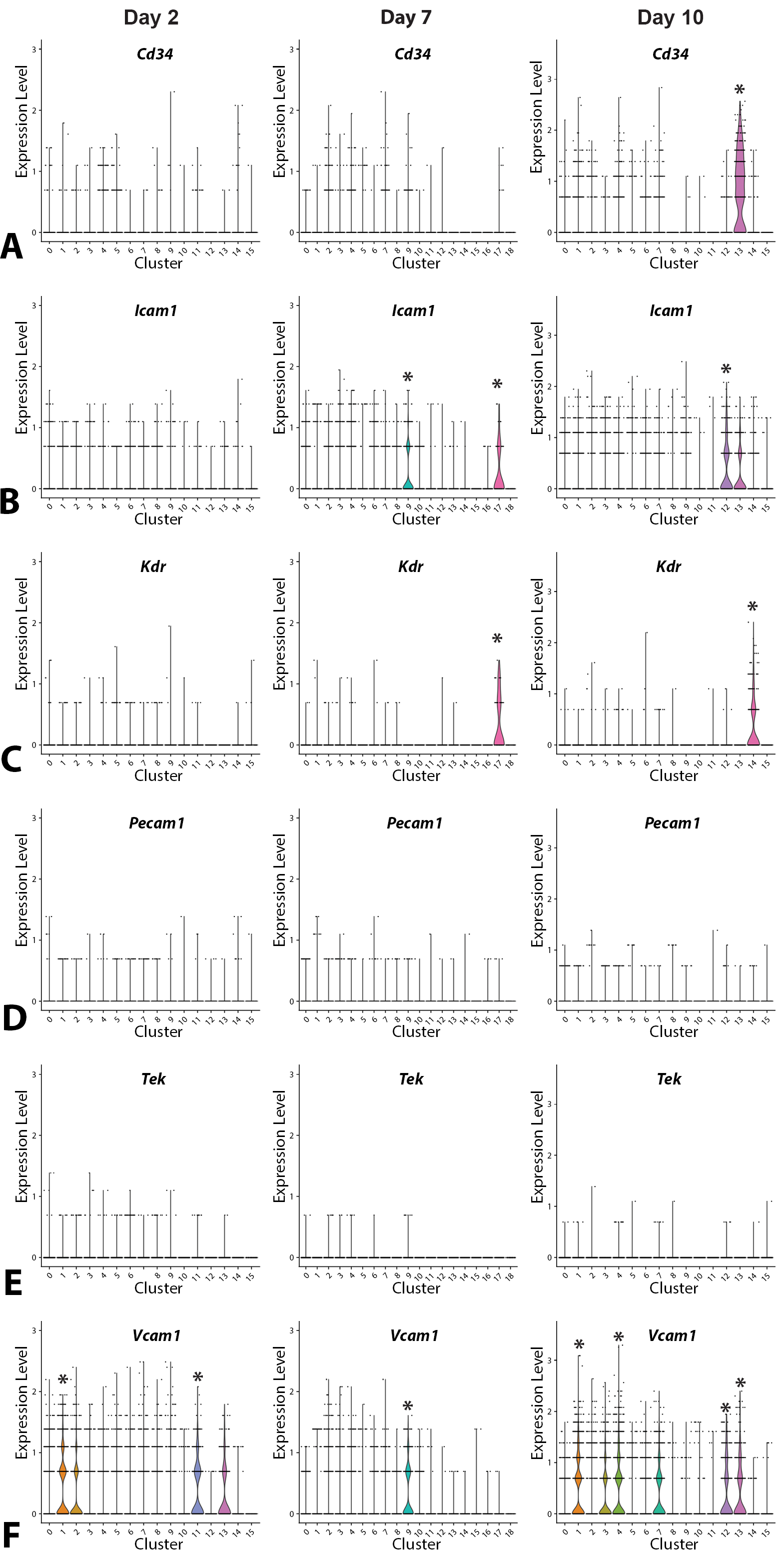

Supplement: Supplementary file 1 [file DataSheet1.zip › Supplementary Data/Supplementary Figure 6.TIF]

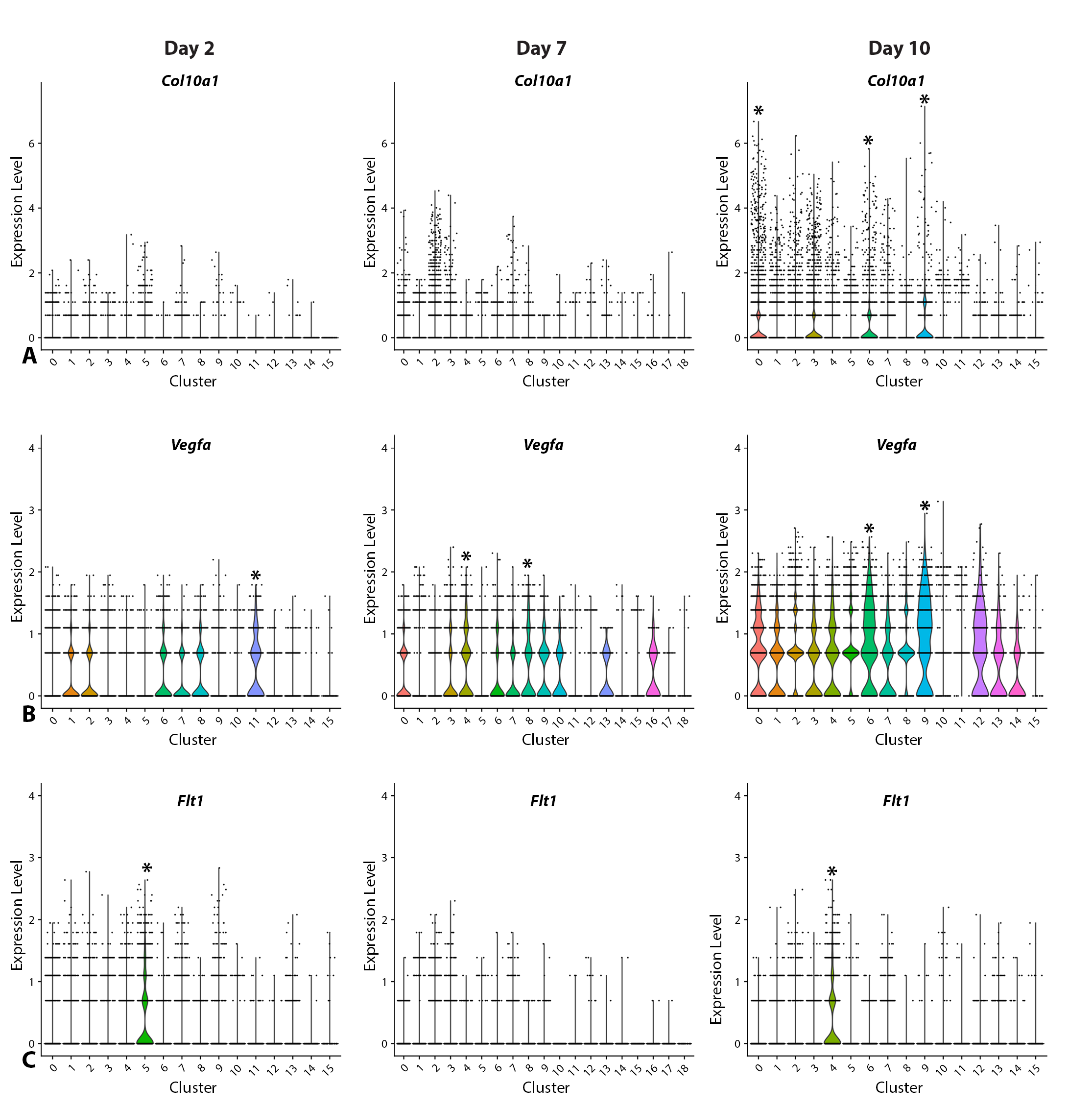

Supplement: Supplementary file 1 [file DataSheet1.zip › Supplementary Data/Supplementary Figure 7.TIF]

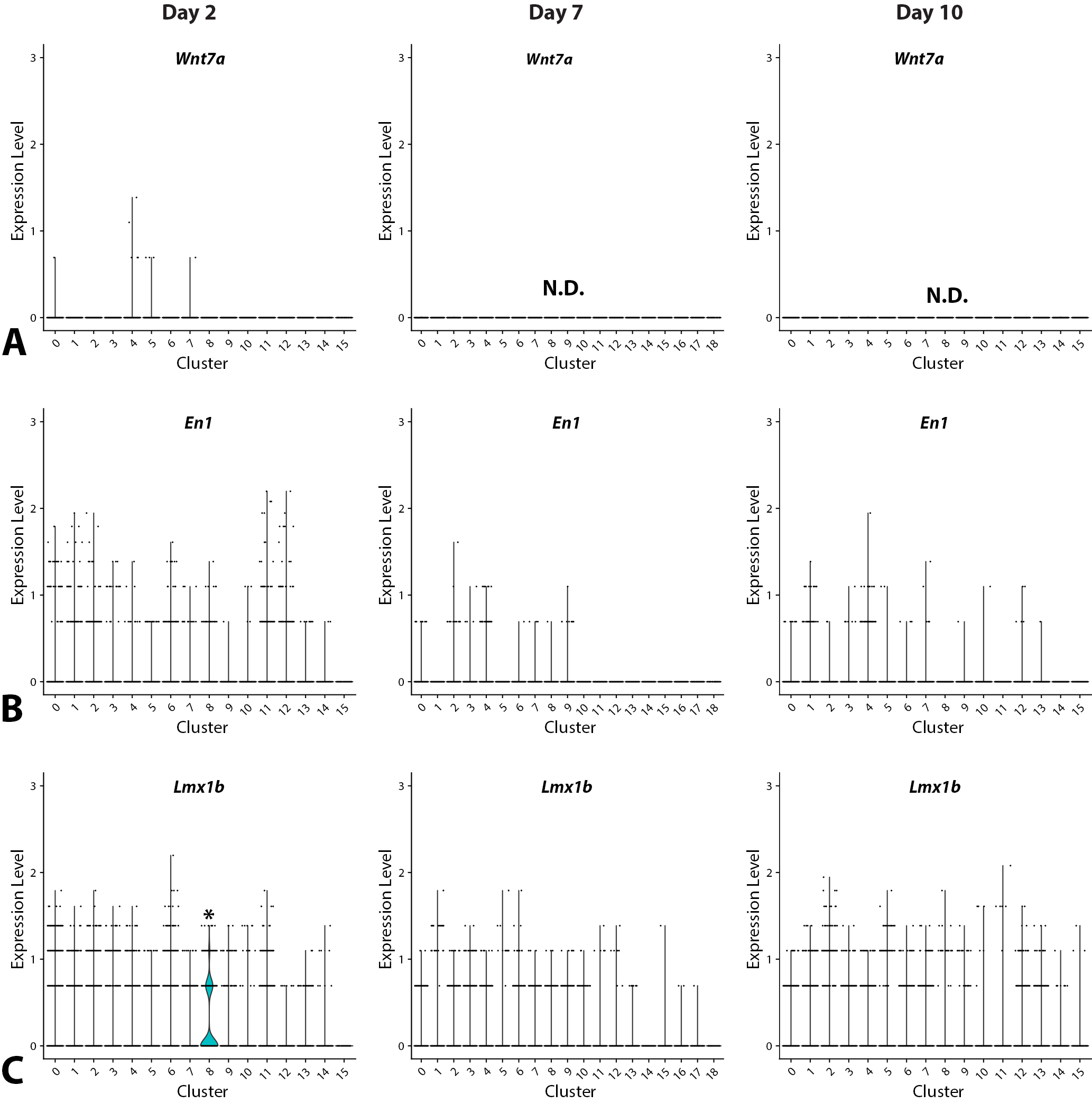

Supplement: Supplementary file 1 [file DataSheet1.zip › Supplementary Data/Supplementary Figure 8.TIF]

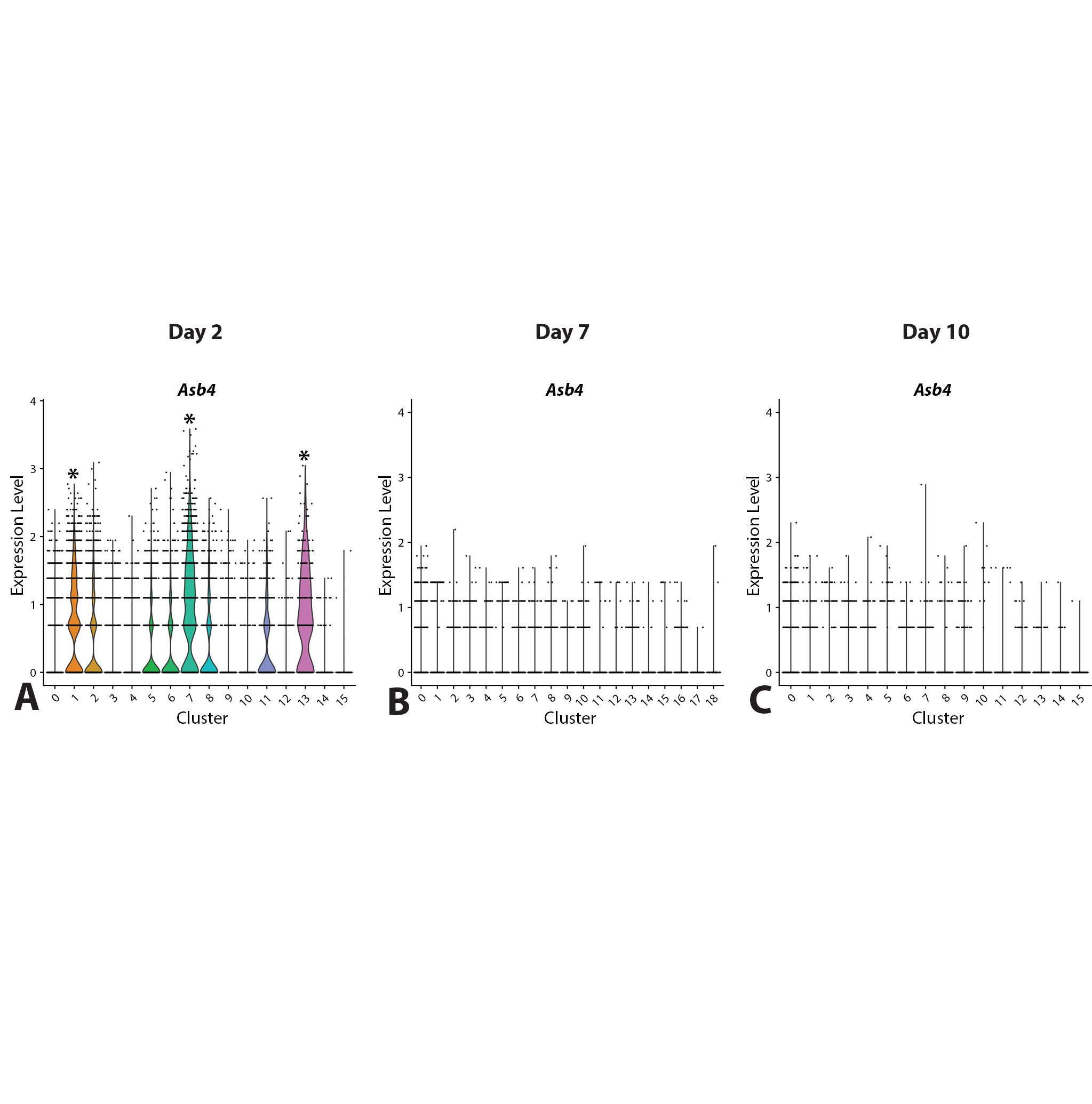

Supplement: Supplementary file 1 [file DataSheet1.zip › Supplementary Data/Supplementary Figure 9.TIF]
